# Supplementary material for: A study of deep active learning methods to reduce labelling efforts in biomedical relation extraction
Source: PLoS One. 2023 Dec 15;18(12):e0292356. doi: 10.1371/journal.pone.0292356 (PMC10723703; doi:10.1371/journal.pone.0292356)
Supplement: S1 File — (PDF) [file pone.0292356.s001.pdf]

Supplementary File 1 for

A Study of Deep Active Learning Methods to Reduce Labelling Efforts in Biomedical Relation Extraction

Charlotte Nachtegael, Jacopo De Stefani, Tom Lenaerts

Corresponding author:
Charlotte Nachtegael: Charlotte.Nachtegael@ulb.be

This document file includes:
Supplementary Figures 1 to 7

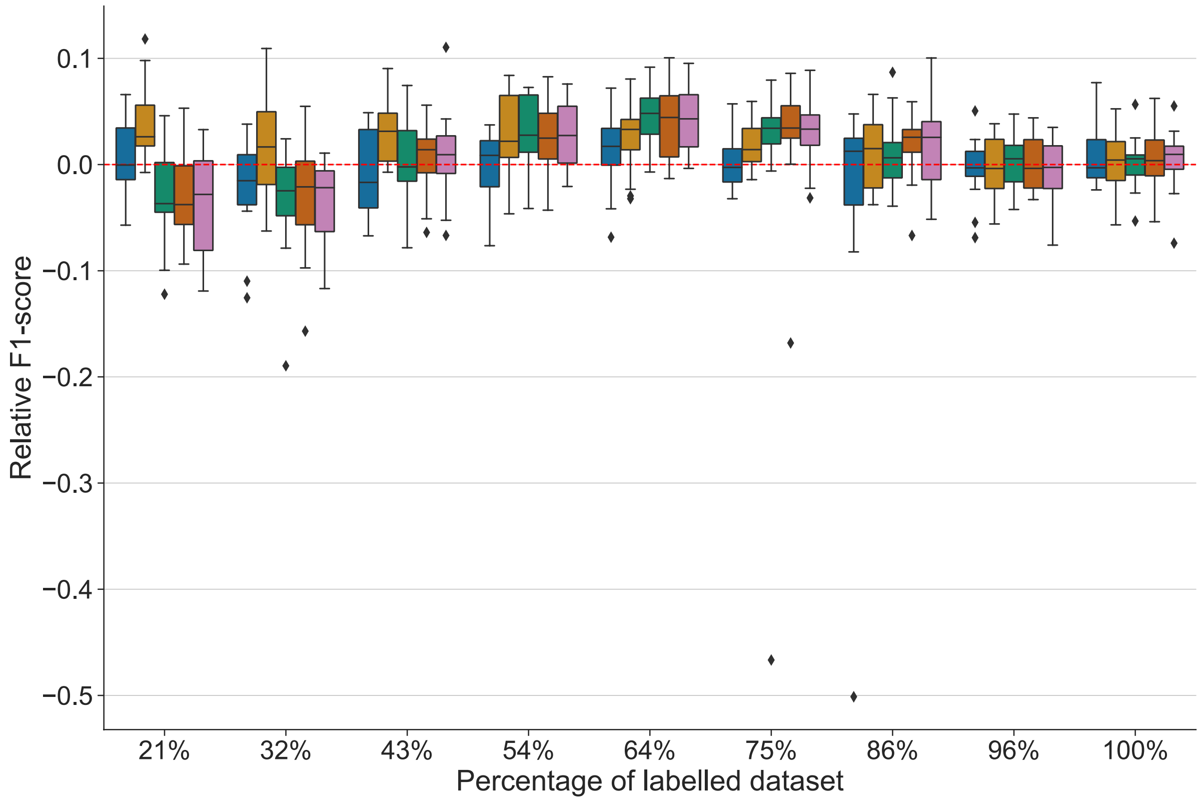

S1 Fig. Boxplot of the relative difference of the F1-score between the AL strategies and the random baseline for the AIMED data set with outliers.

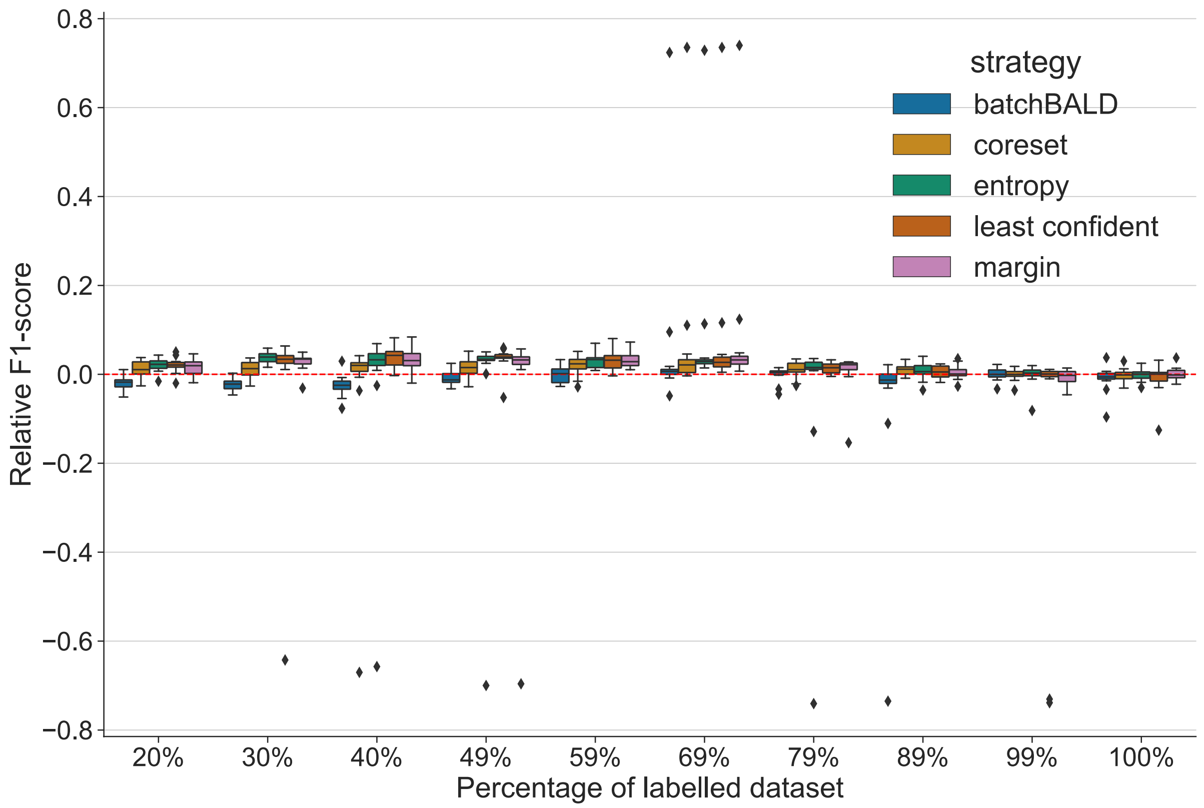

S2 Fig. Boxplot of the relative difference of the F1-score between the AL strategies and the random baseline for the BioRED data set with outliers.

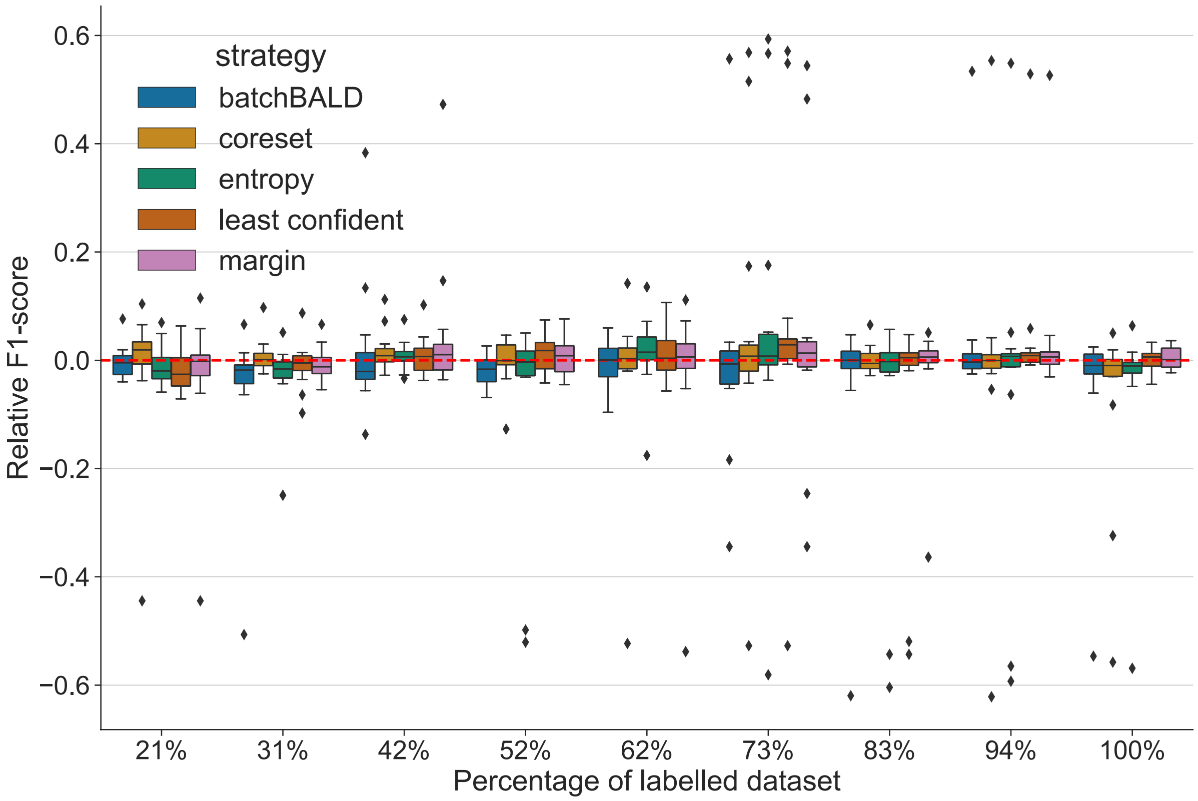

S3 Fig. Boxplot of the relative difference of the F1-score between the AL strategies and the random baseline for the CDR data set with outliers.

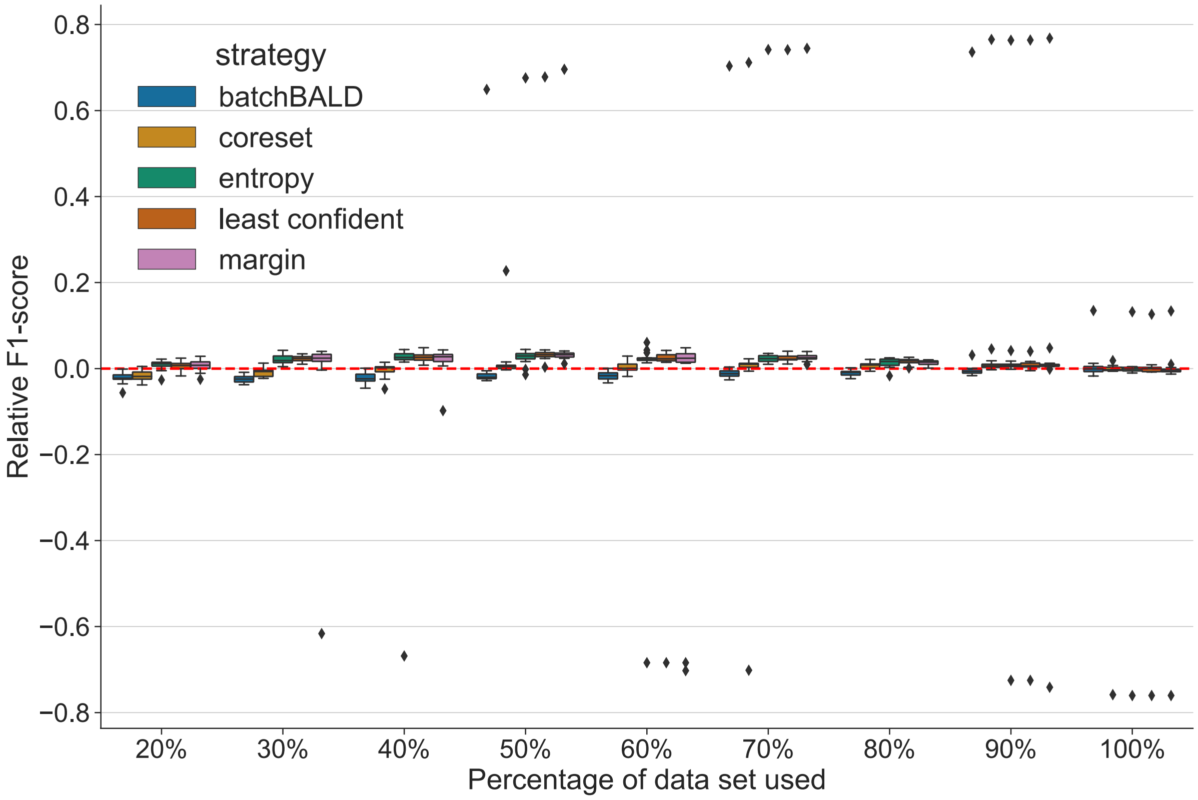

S4 Fig. Boxplot of the relative difference of the F1-score between the AL strategies and the random baseline for the ChemProt data set with outliers.

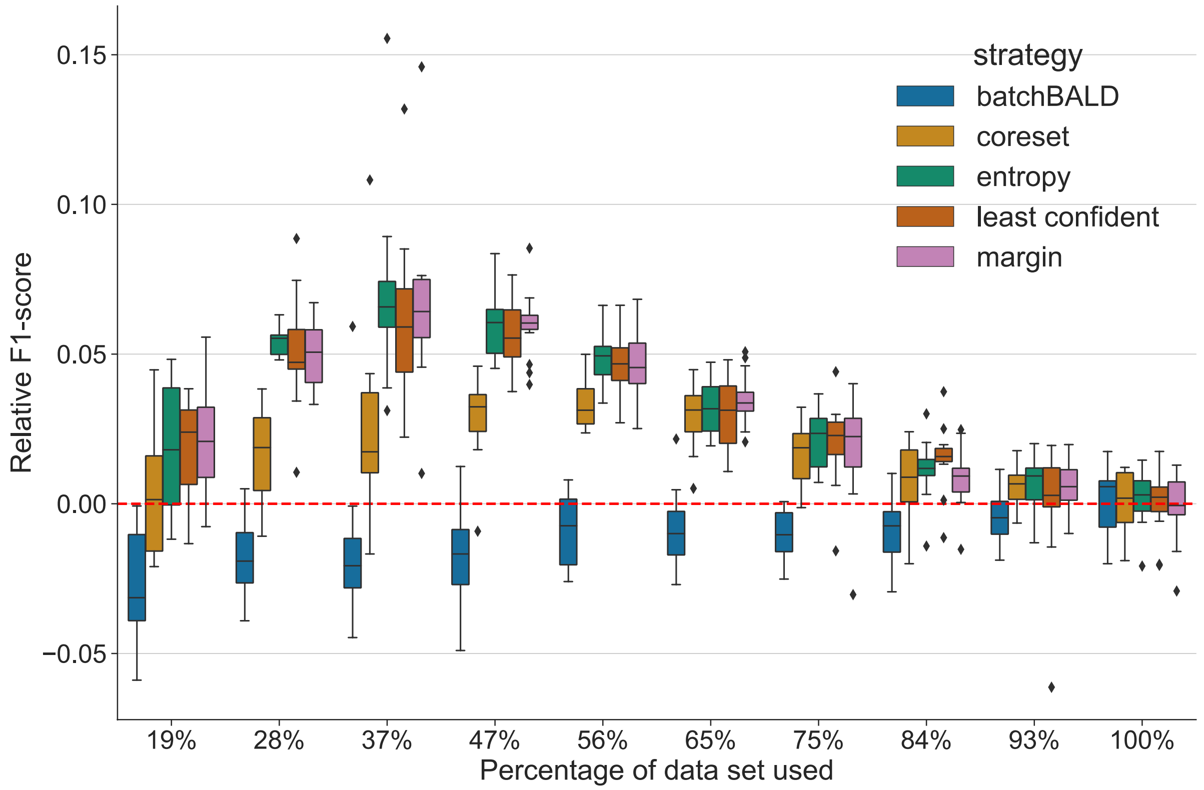

S5 Fig. Boxplot of the relative difference of the F1-score between the AL strategies and the random baseline for the DDI data set with outliers.

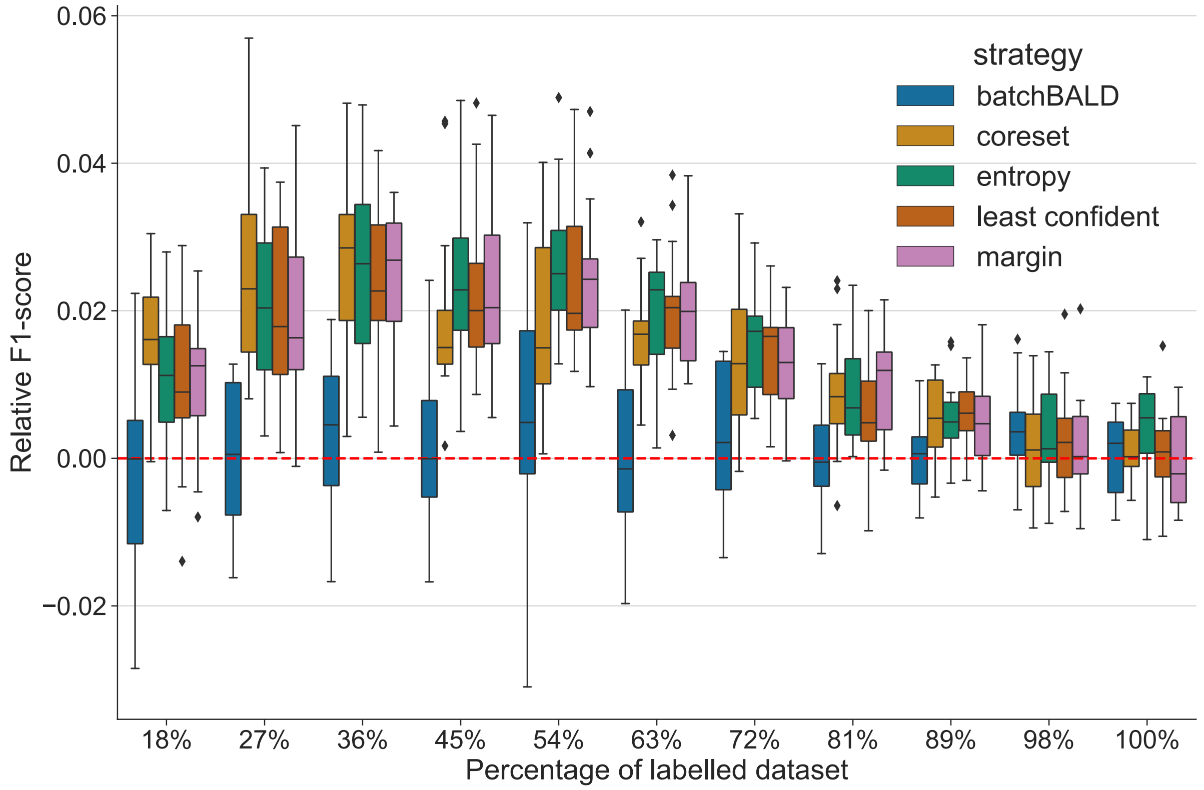

S6 Fig. Boxplot of the relative difference of the F1-score between the AL strategies and the random baseline for the Nary-DGV data set with outliers.

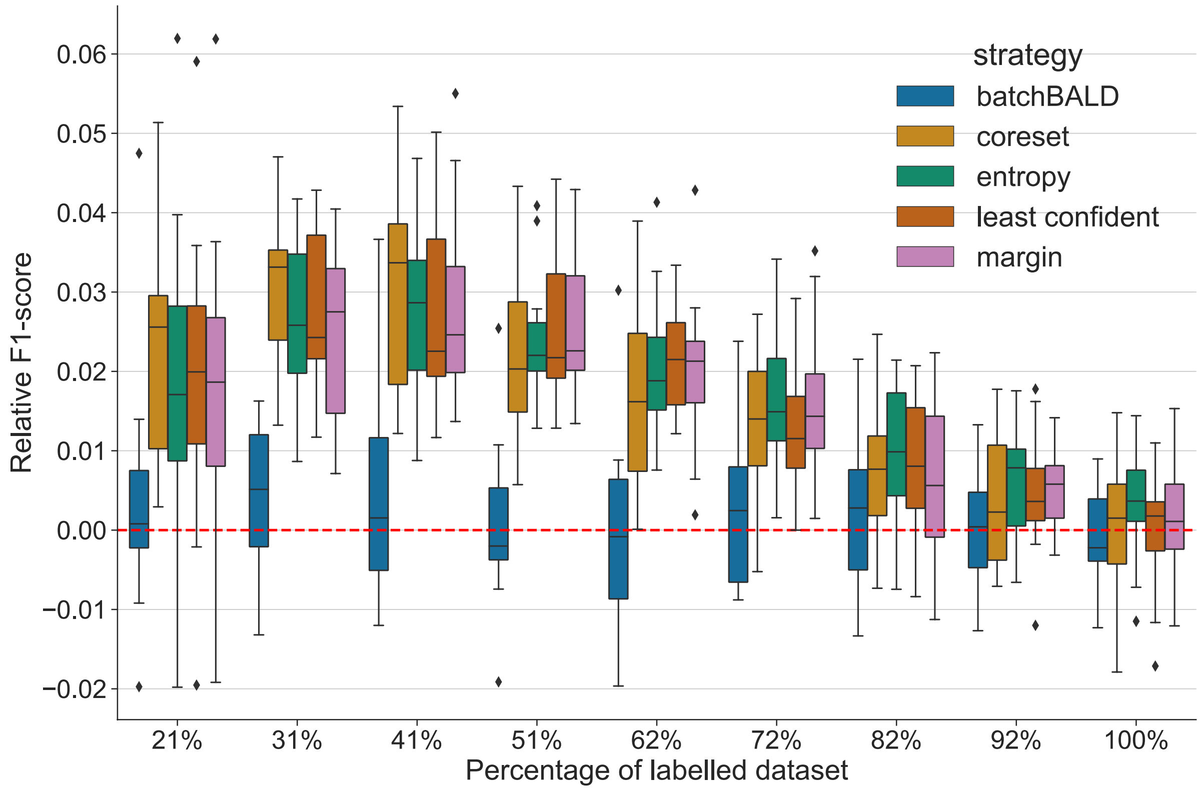

S7 Fig. Boxplot of the relative difference of the F1-score between the AL strategies and the random baseline for the Nary-DV data set with outliers.
