# Supplementary material for: A study of deep active learning methods to reduce labelling efforts in biomedical relation extraction
Source: PLoS One. 2023 Dec 15;18(12):e0292356. doi: 10.1371/journal.pone.0292356 (PMC10723703; doi:10.1371/journal.pone.0292356)
Supplement: S2 File — (PDF) [file pone.0292356.s002.pdf]

Supplementary File 2 for

A Study of Deep Active Learning Methods to Reduce Labelling Efforts in Biomedical Relation Extraction

Charlotte Nachtegael, Jacopo De Stefani, Tom Lenaerts

Corresponding author:
Charlotte Nachtegael: Charlotte.Nachtegael@ulb.be

This PDF file includes:
Supplementary Figures 8 to 35

Supplementary Figures

AIMED

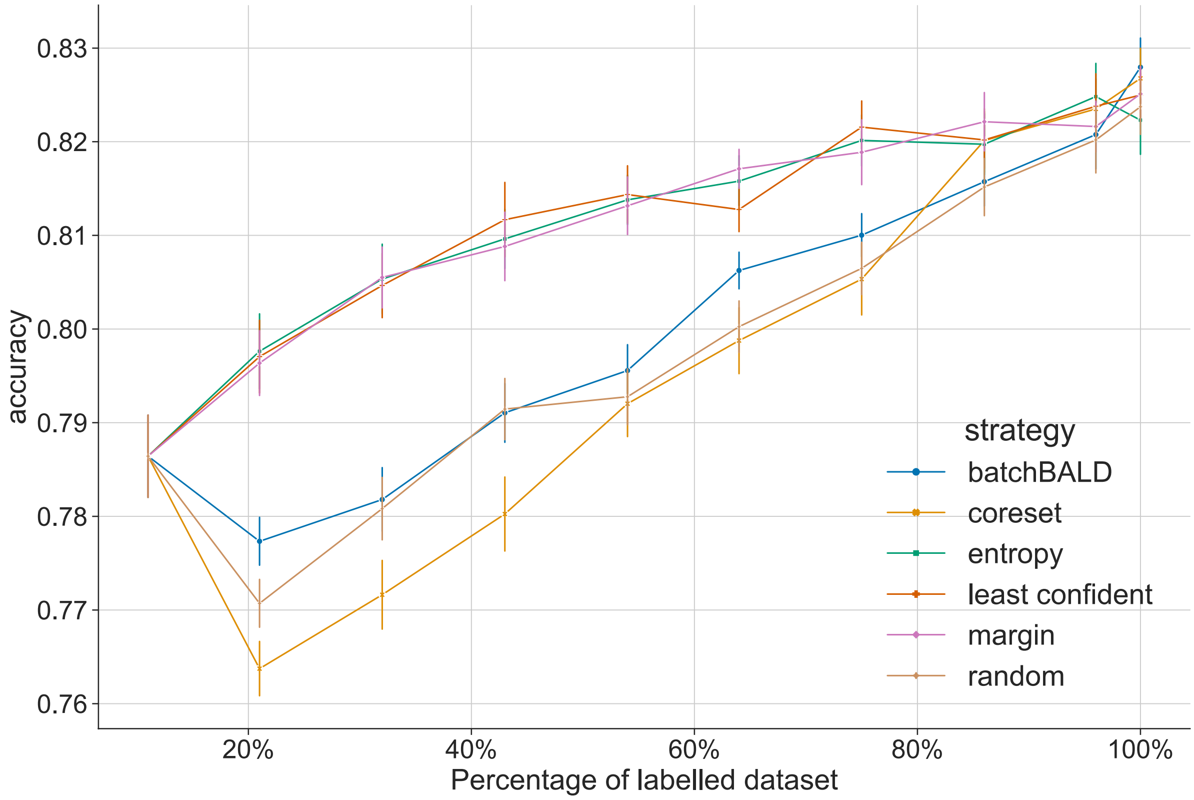

S8 Fig. Accuracy over the active learning iterations for the AIMED data set. Error bars correspond to the standard error.

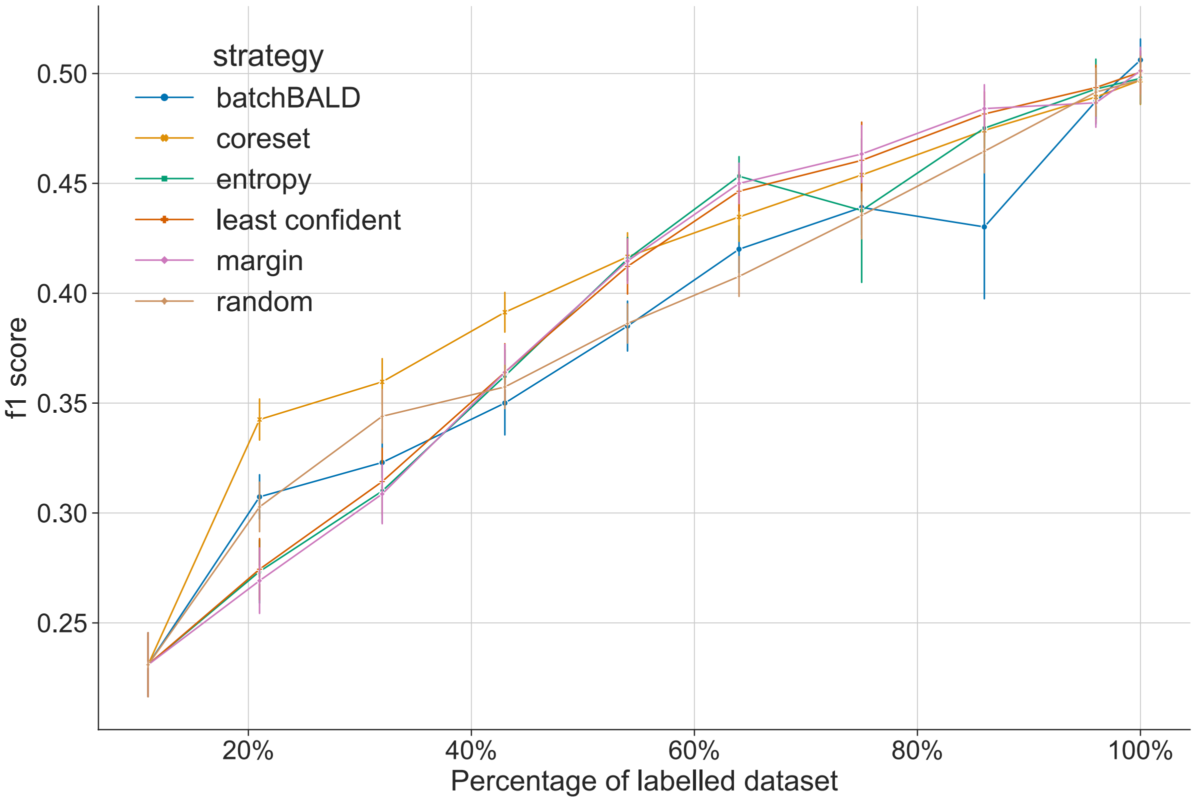

S9 Fig. F1-score over the active learning iterations for the AIMED data set. Error bars correspond to the standard error.

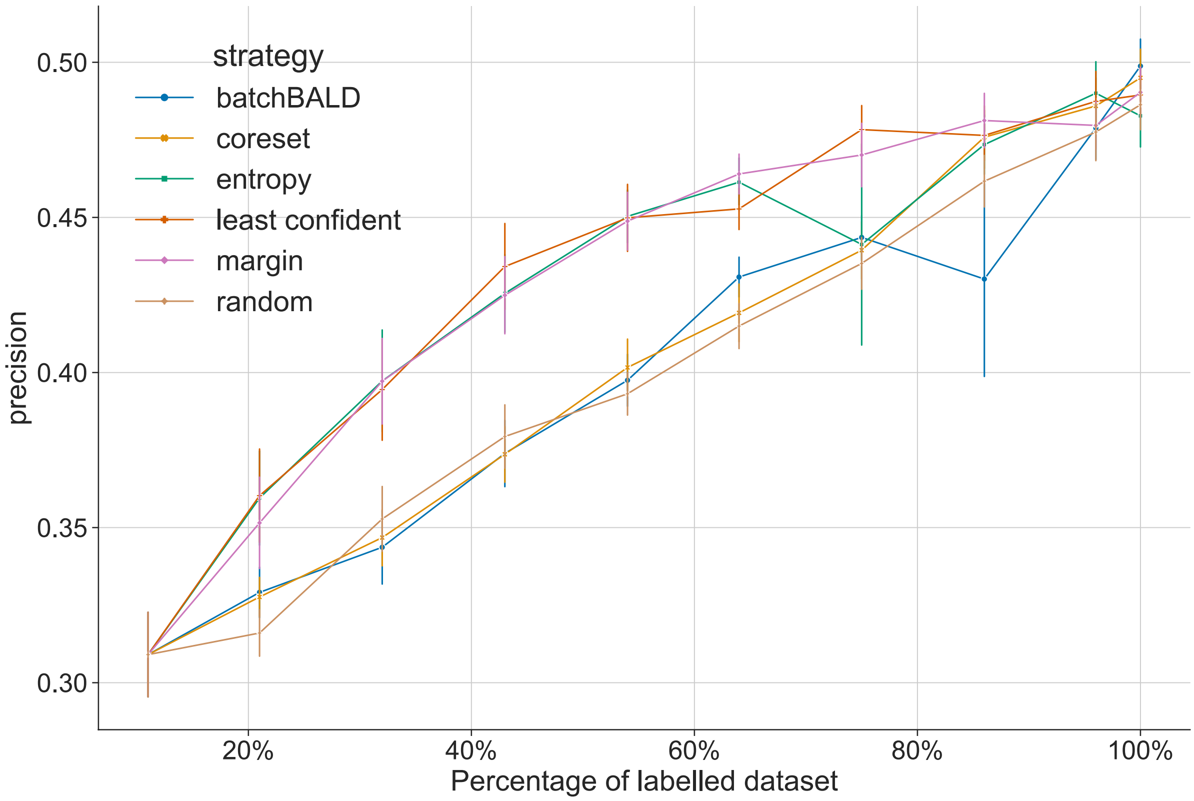

S10 Fig. Precision over the active learning iterations for the AIMED data set. Error bars correspond to the standard error.

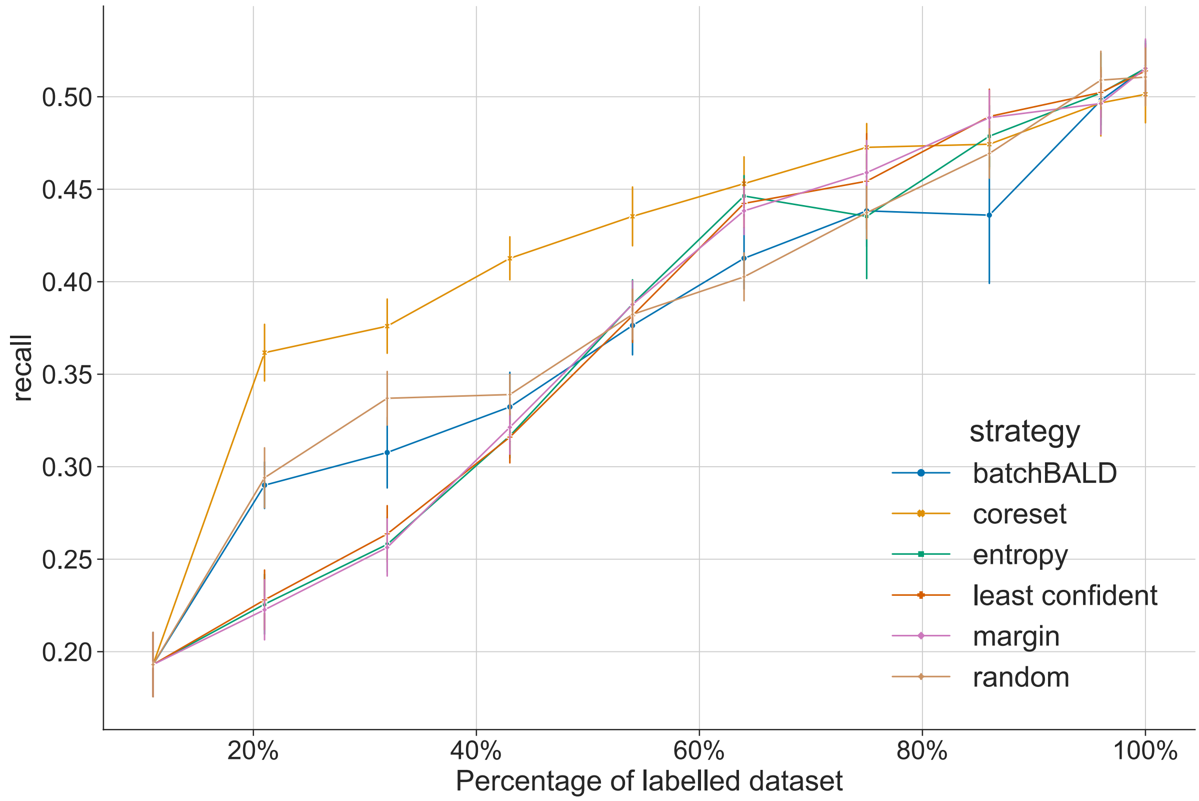

S11 Fig. Recall over the active learning iterations for the AIMED data set. Error bars correspond to the standard error.

BioRED

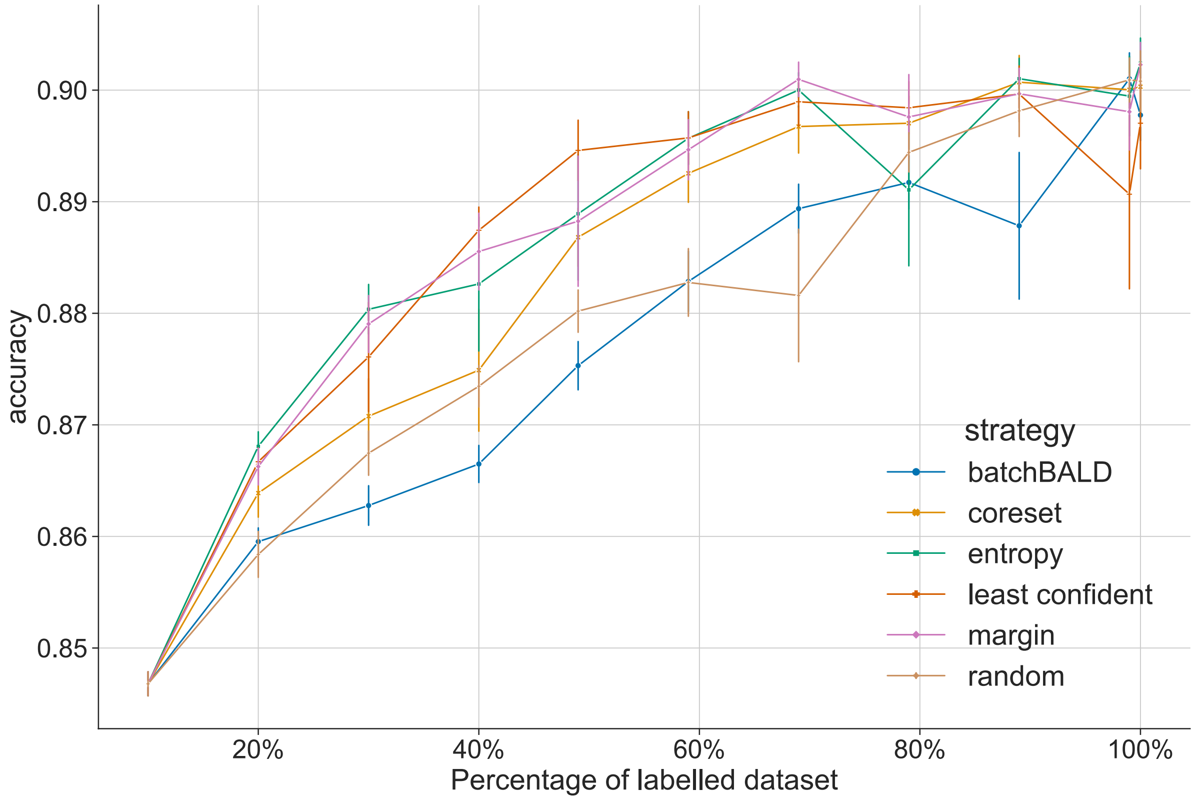

S12 Fig. Accuracy over the active learning iterations for the BioRED data set. Error bars correspond to the standard error.

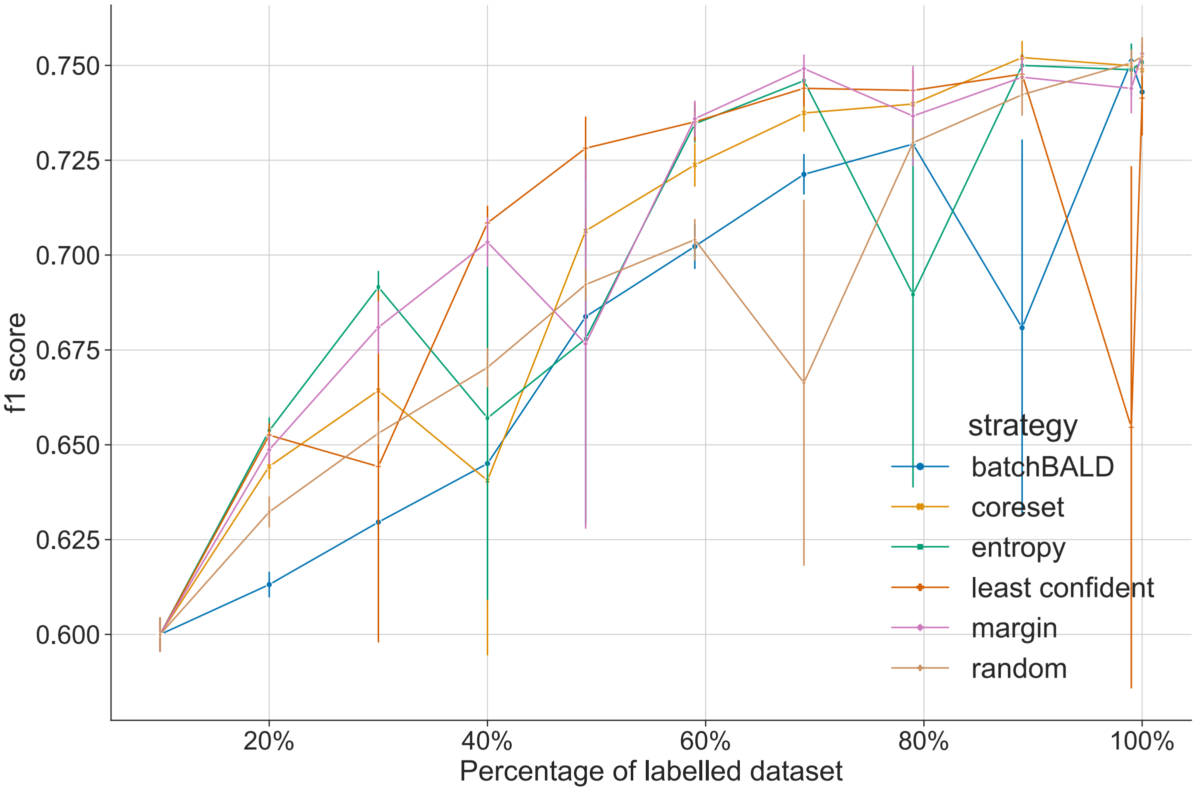

S13 Fig. F1-score over the active learning iterations for the BioRED data set. Error bars correspond to the standard error.

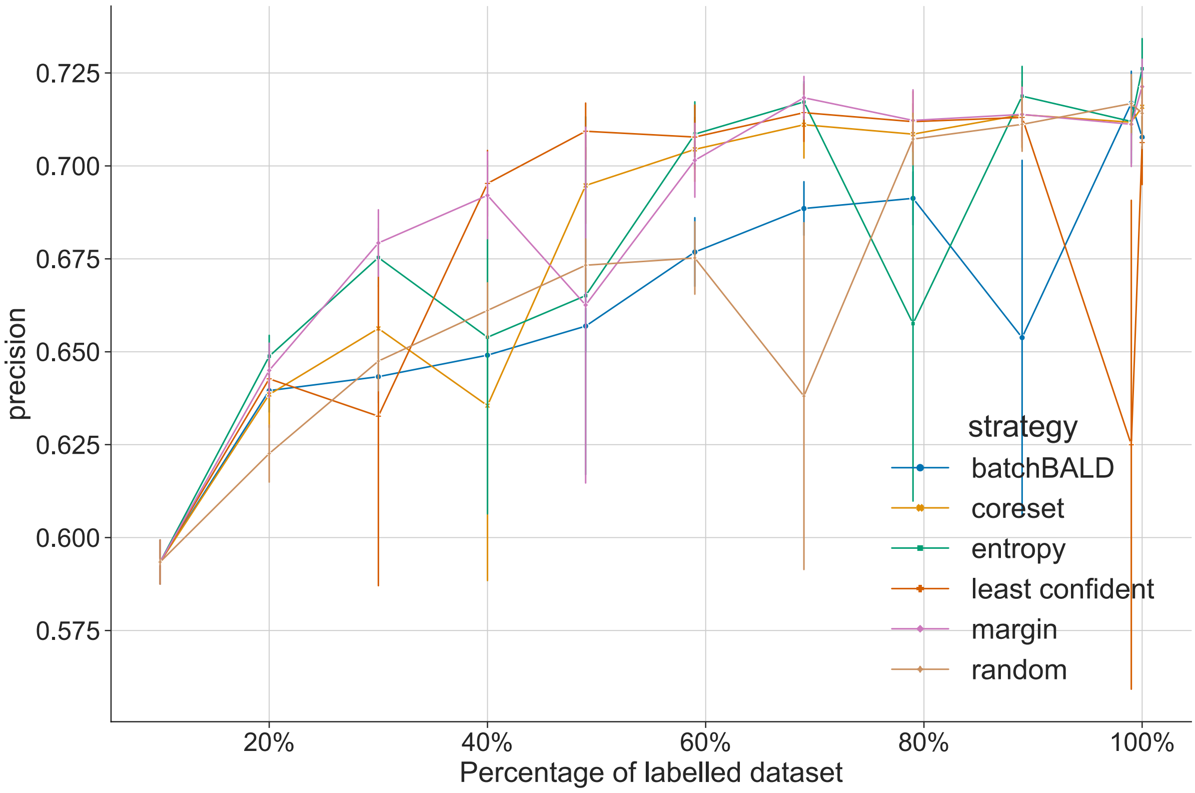

S14 Fig. Precision over the active learning iterations for the BioRED data set. Error bars correspond to the standard error.

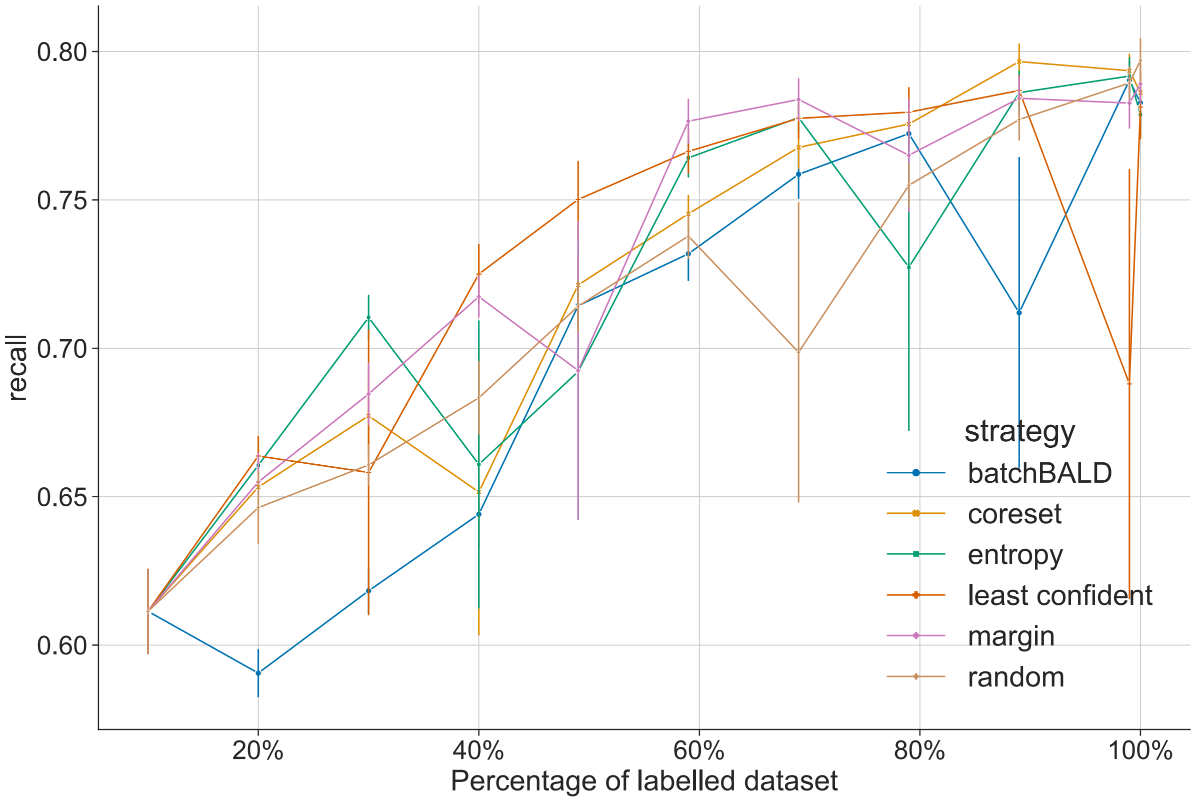

S15 Fig. Recall over the active learning iterations for the BioRED data set. Error bars correspond to the standard error.

CDR

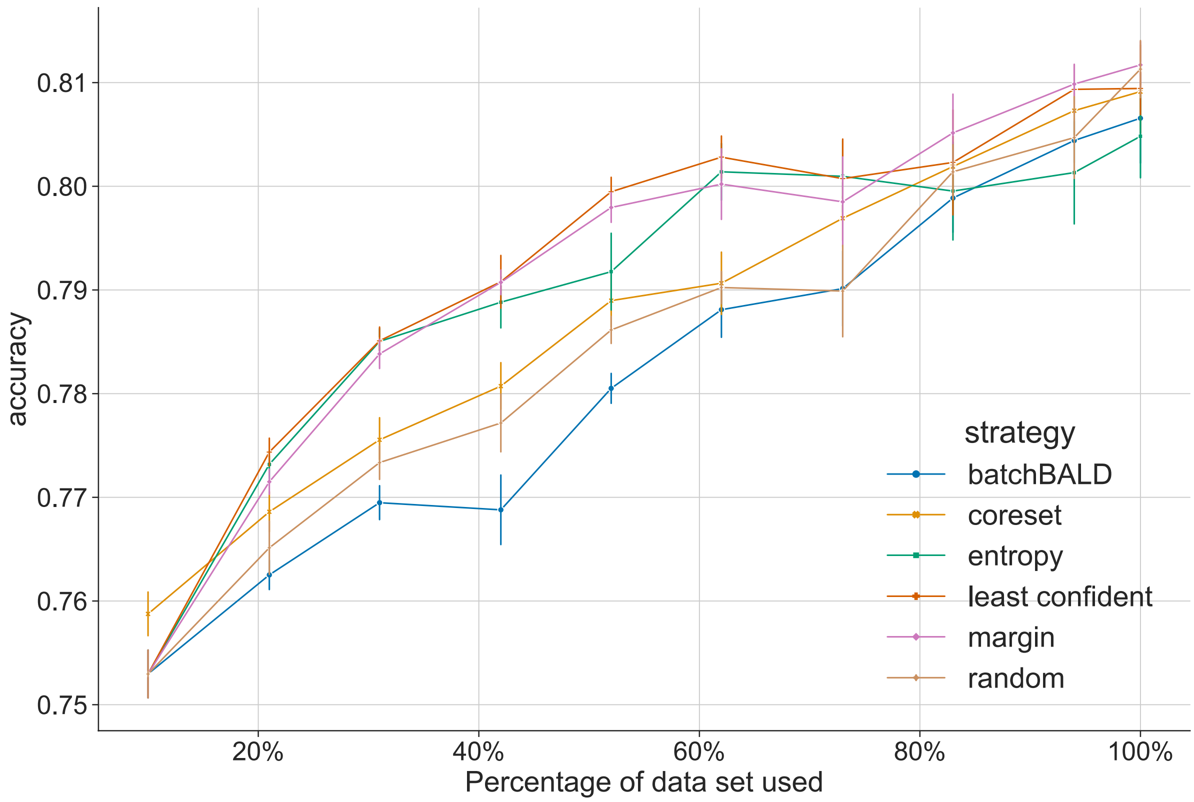

S16 Fig. Accuracy over the active learning iterations for the CDR data set. Error bars correspond to the standard error.

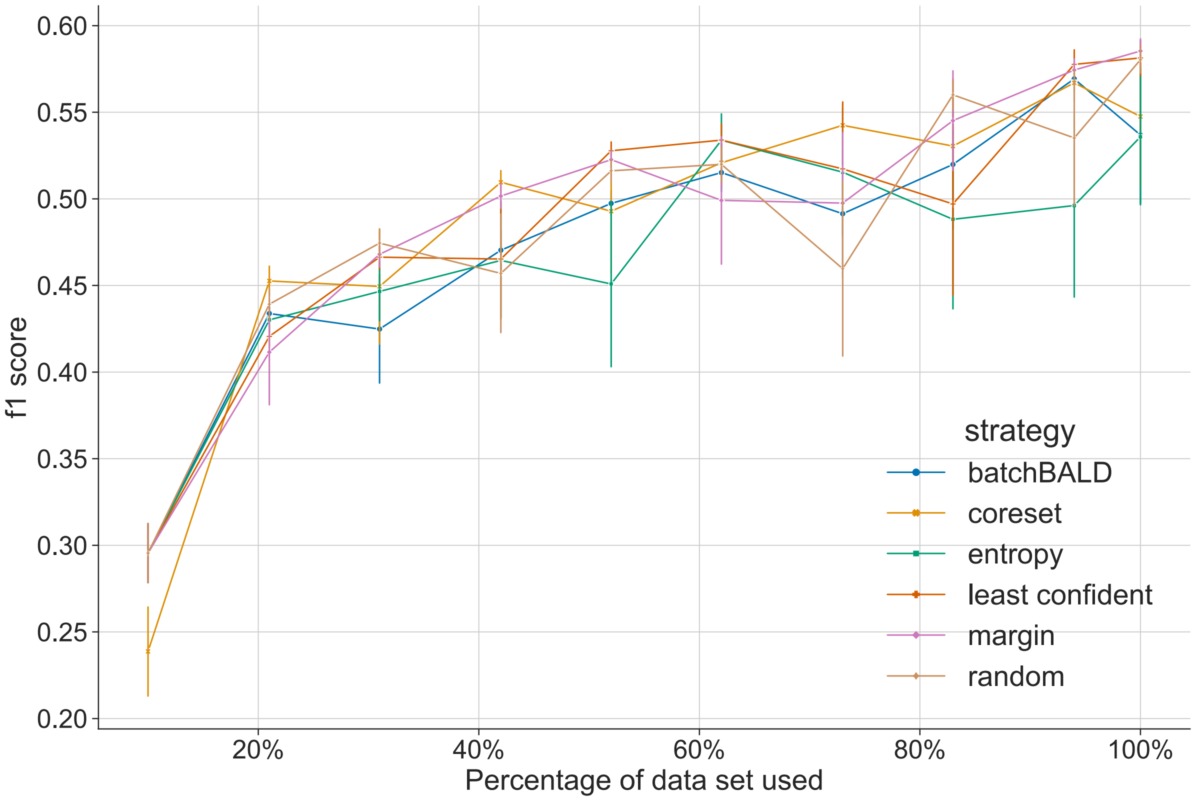

S17 Fig. F1-score over the active learning iterations for the CDR data set. Error bars correspond to the standard error.

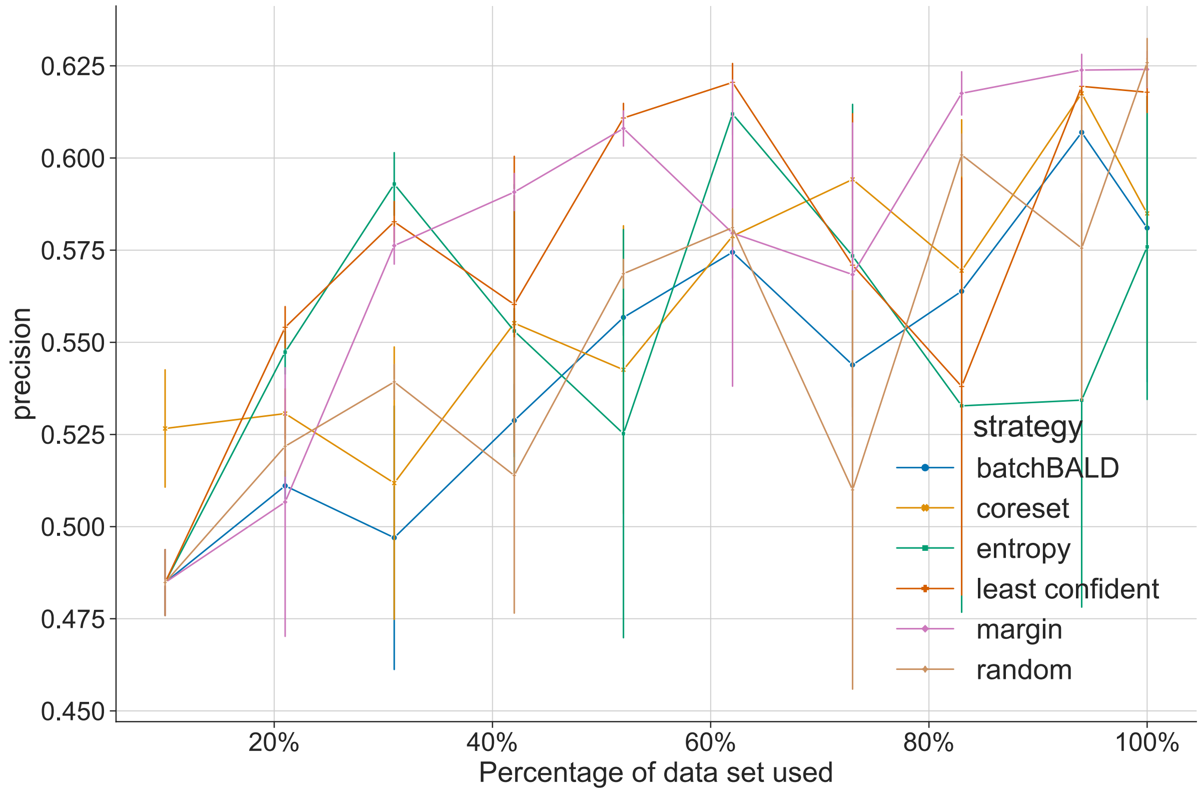

S18 Fig. Precision over the active learning iterations for the CDR data set. Error bars correspond to the standard error.

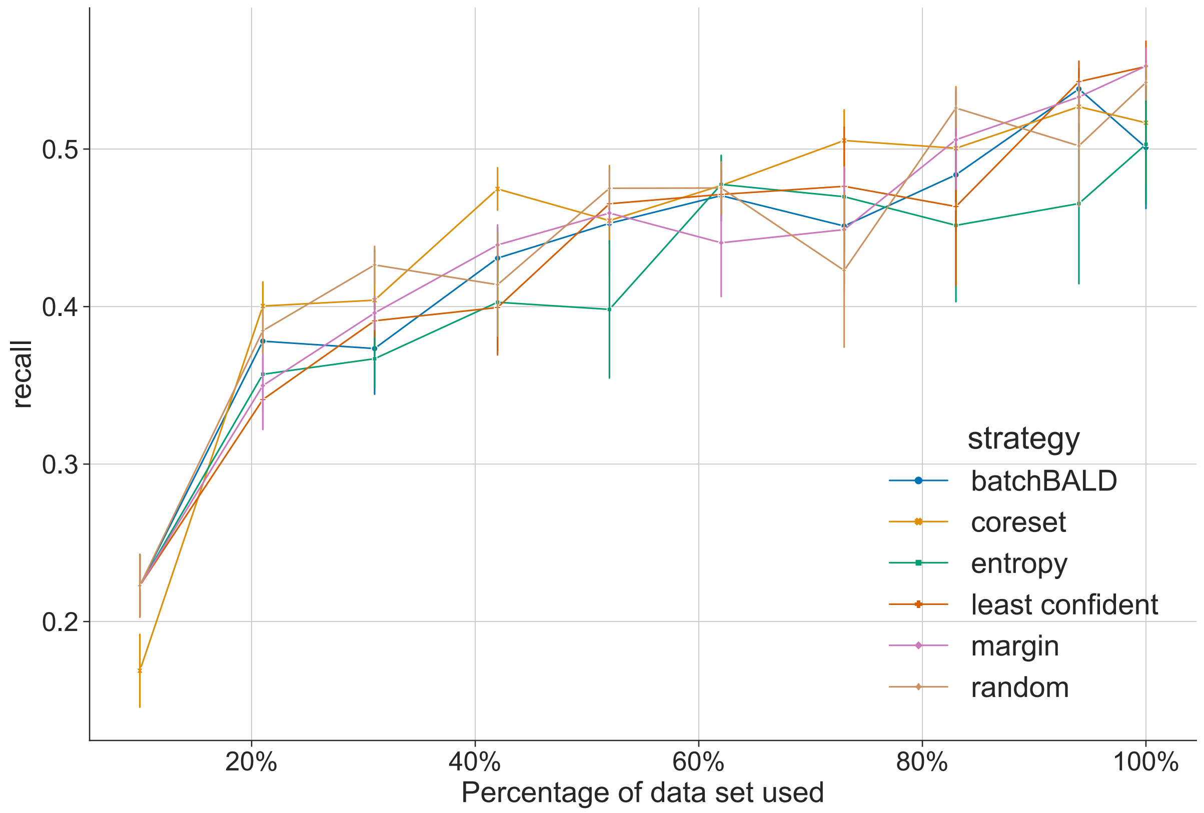

S19 Fig. Recall over the active learning iterations for the CDR data set. Error bars correspond to the standard error.

ChemProt

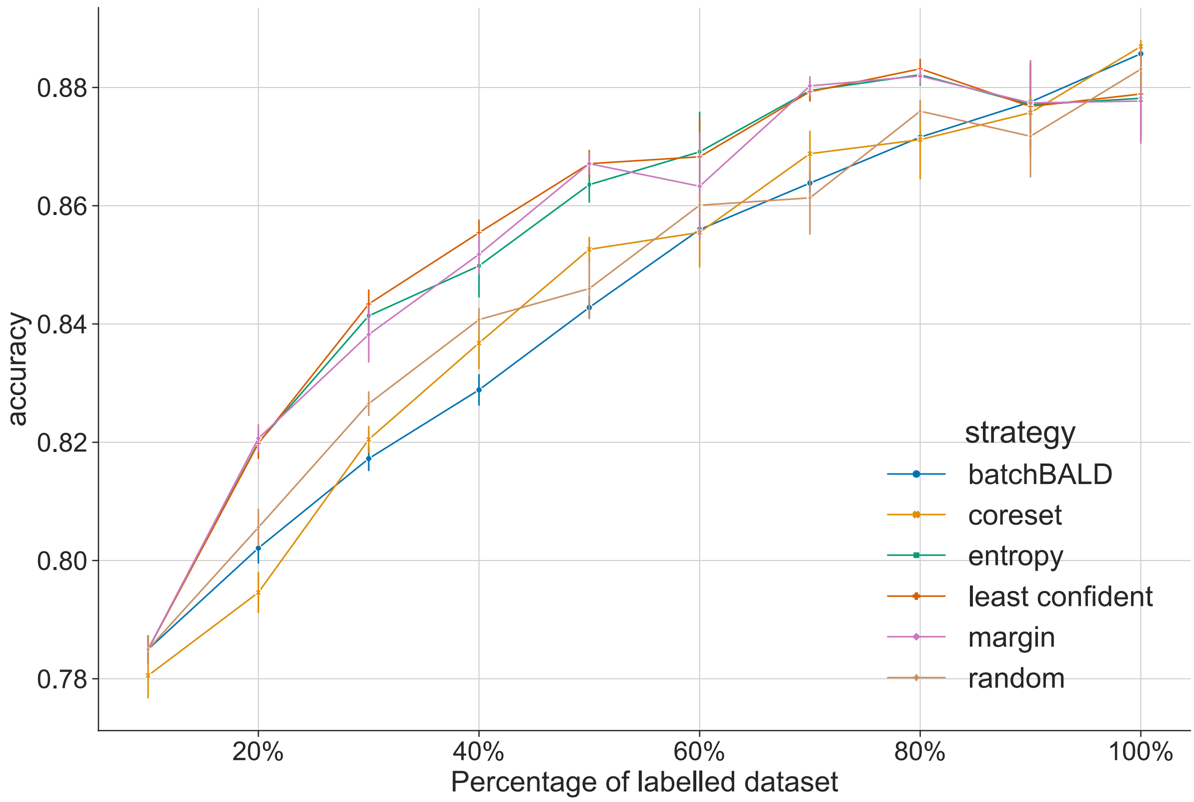

S20 Fig. Accuracy over the active learning iterations for the ChemProt data set. Error bars correspond to the standard error.

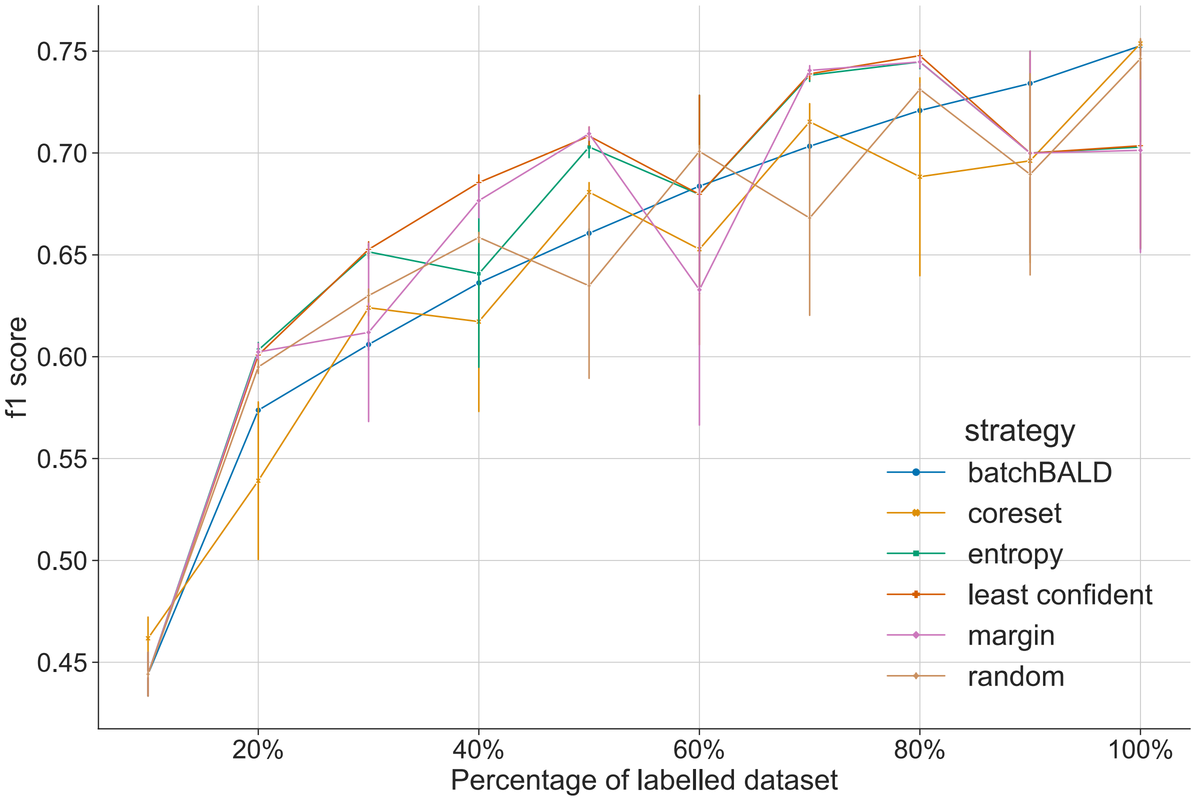

S21 Fig. F1-score over the active learning iterations for the ChemProt data set. Error bars correspond to the standard error.

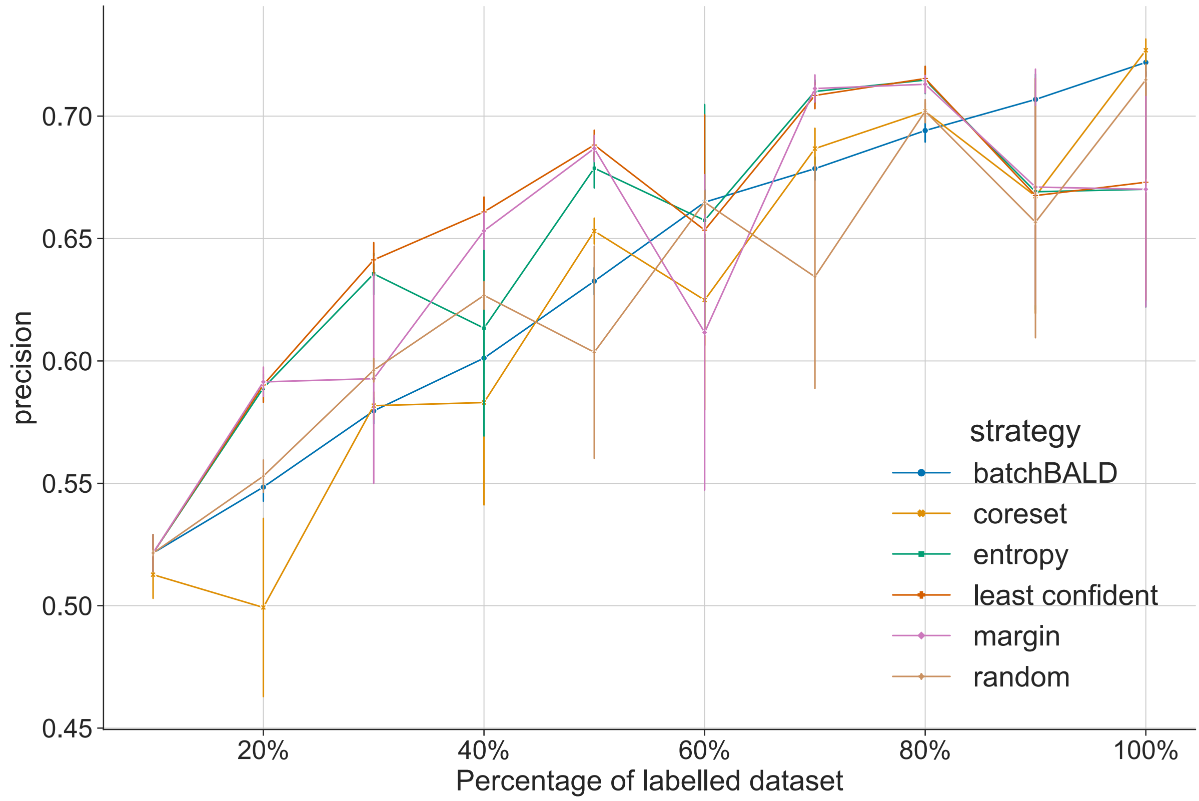

S22 Fig. Precision over the active learning iterations for the ChemProt data set. Error bars correspond to the standard error.

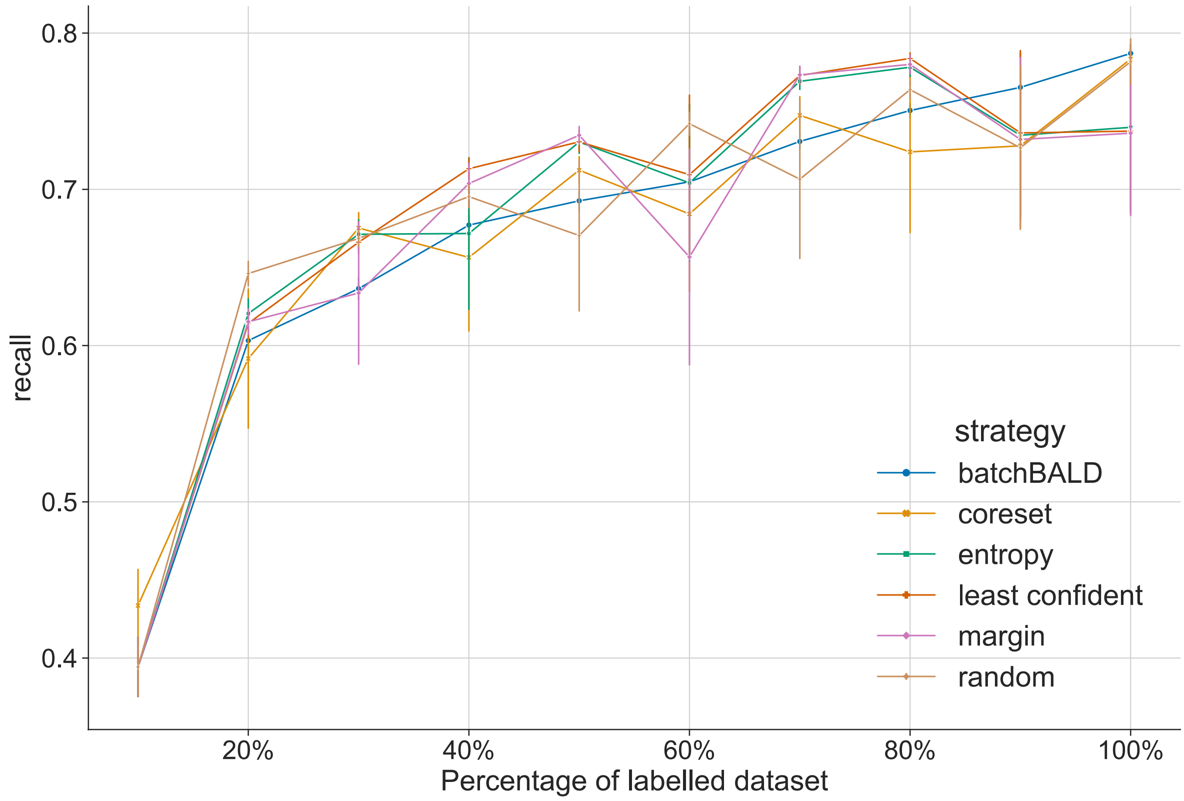

S23 Fig. Recall over the active learning iterations for the ChemProt data set. Error bars correspond to the standard error.

DDI

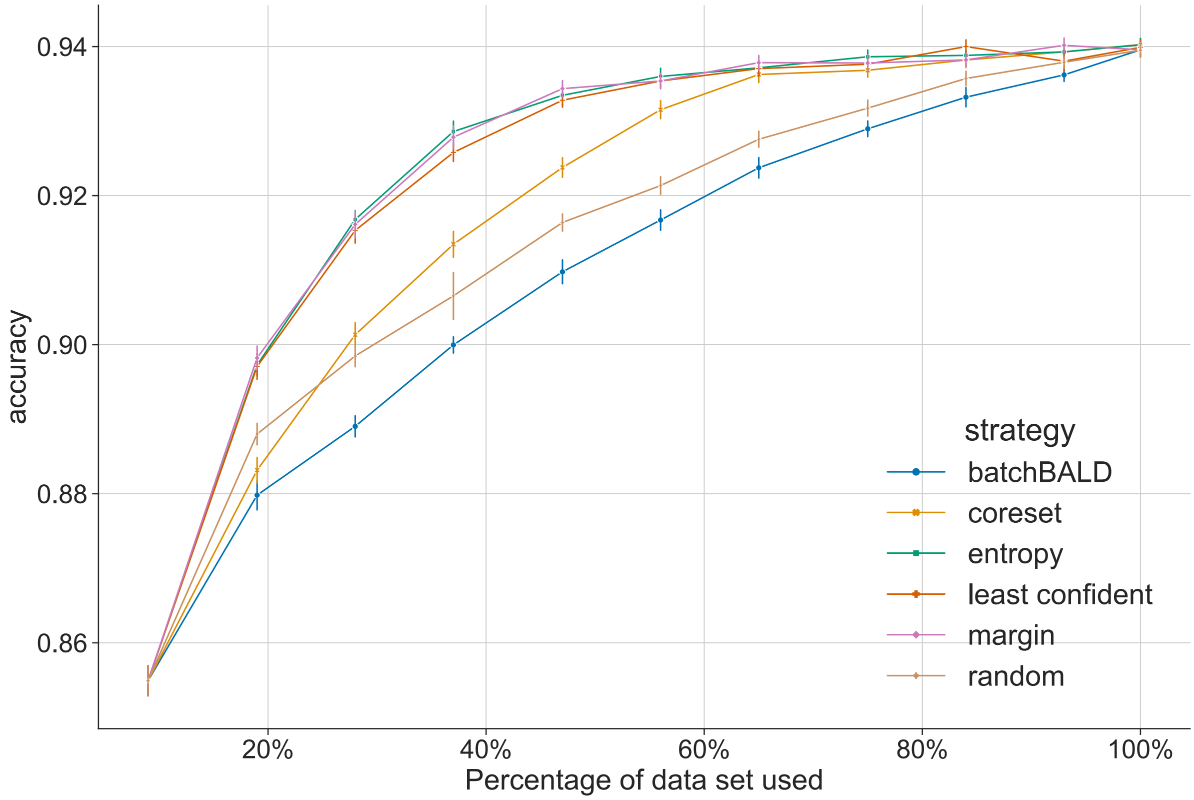

S24 Fig. Accuracy over the active learning iterations for the DDI data set. Error bars correspond to the standard error.

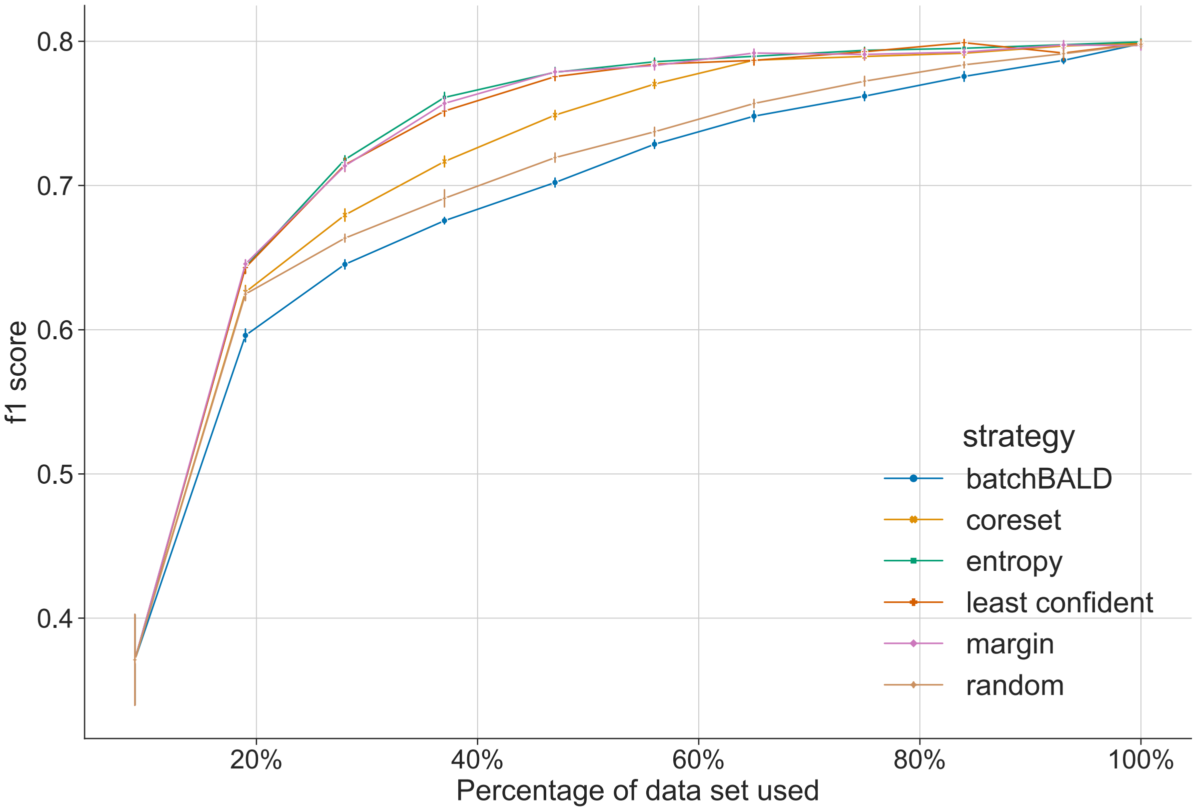

S25 Fig. F1-score over the active learning iterations for the DDI data set. Error bars correspond to the standard error.

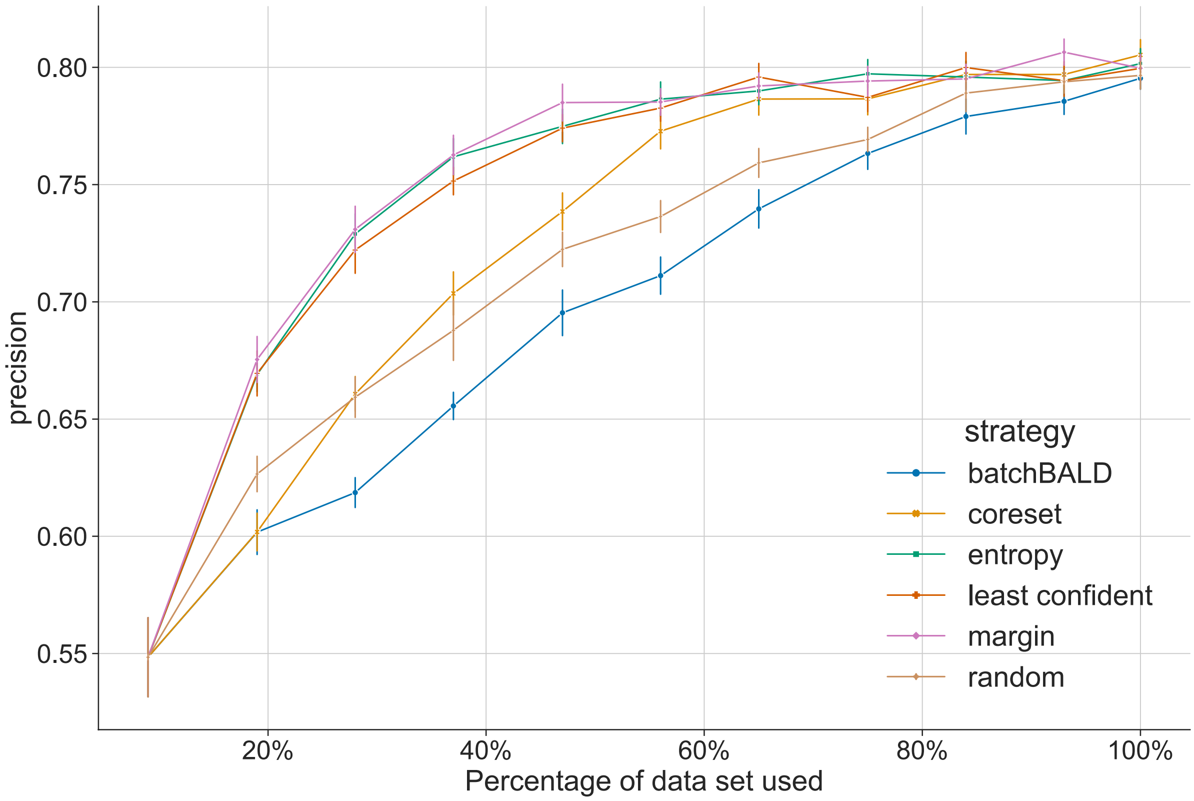

S26 Fig. Precision over the active learning iterations for the DDI data set. Error bars correspond to the standard error.

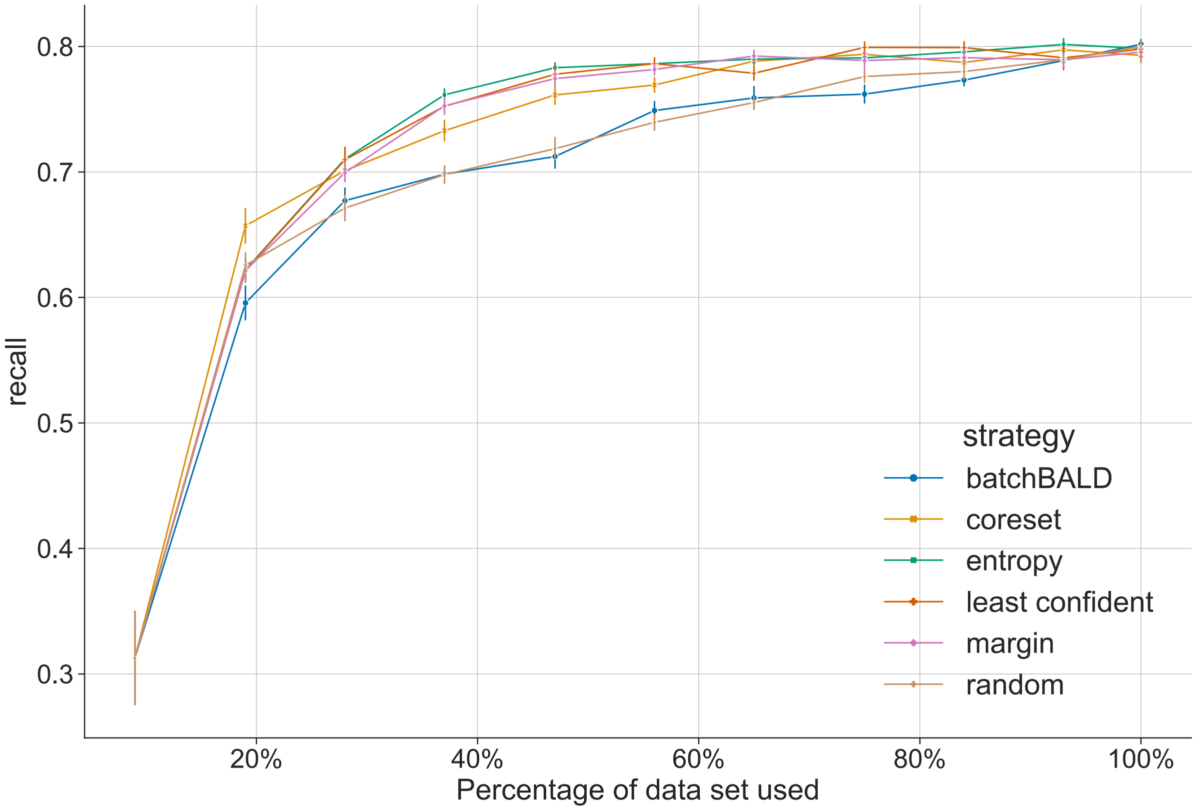

S27 Fig. Recall over the active learning iterations for the DDI data set. Error bars correspond to the standard error.

Nary-DGV

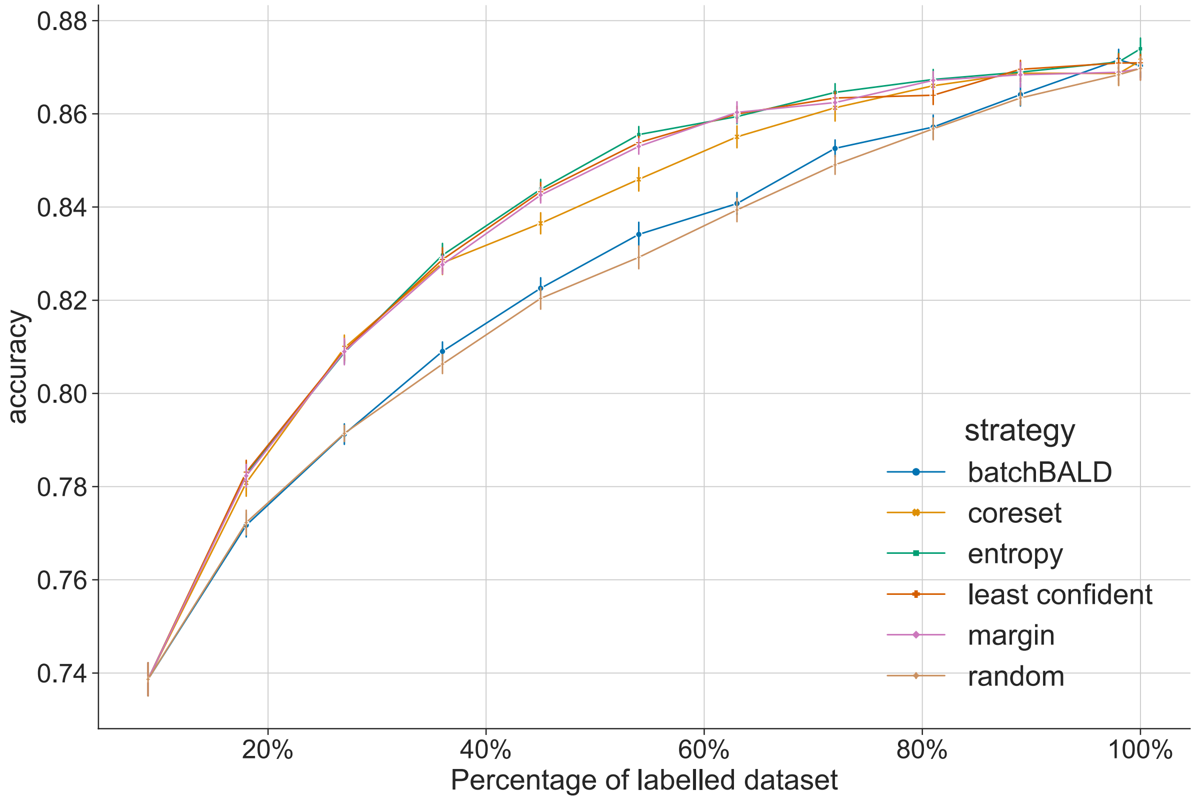

S28 Fig. Accuracy over the active learning iterations for the Nary-DGV data set. Error bars correspond to the standard error.

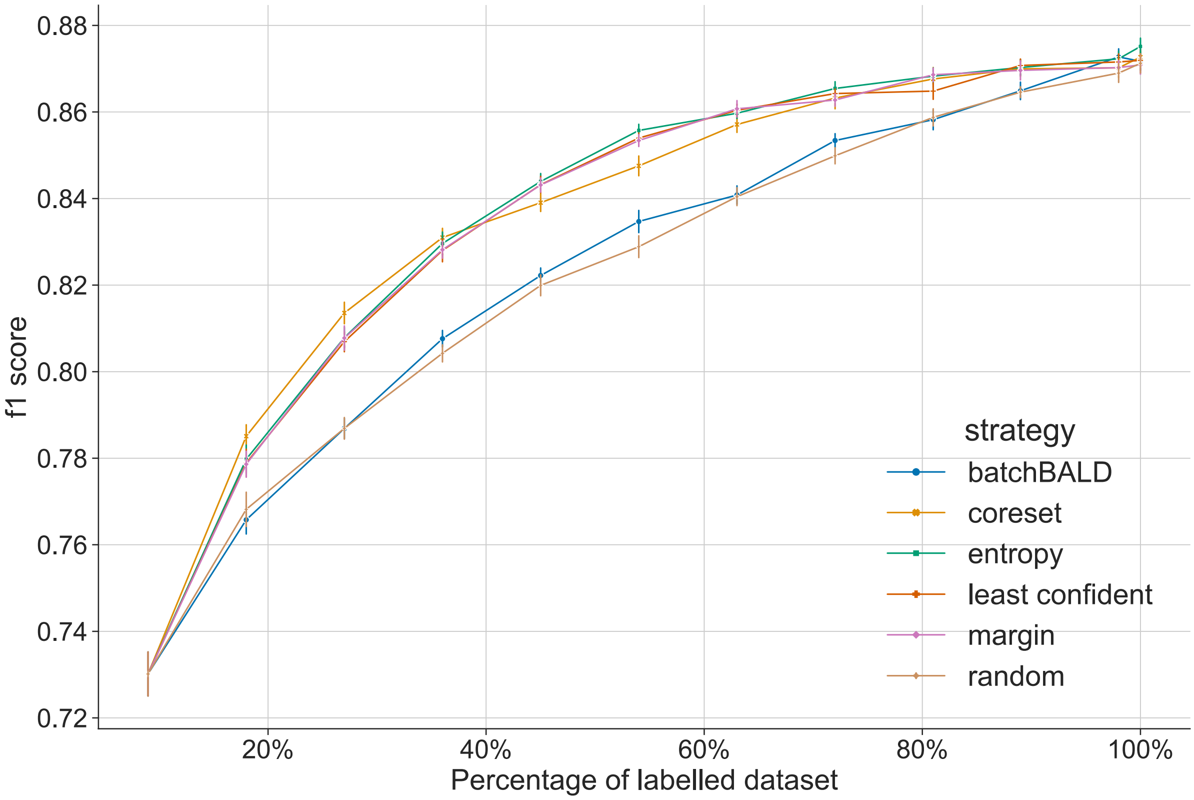

S29 Fig. F1-score over the active learning iterations for the Nary-DGV data set. Error bars correspond to the standard error.

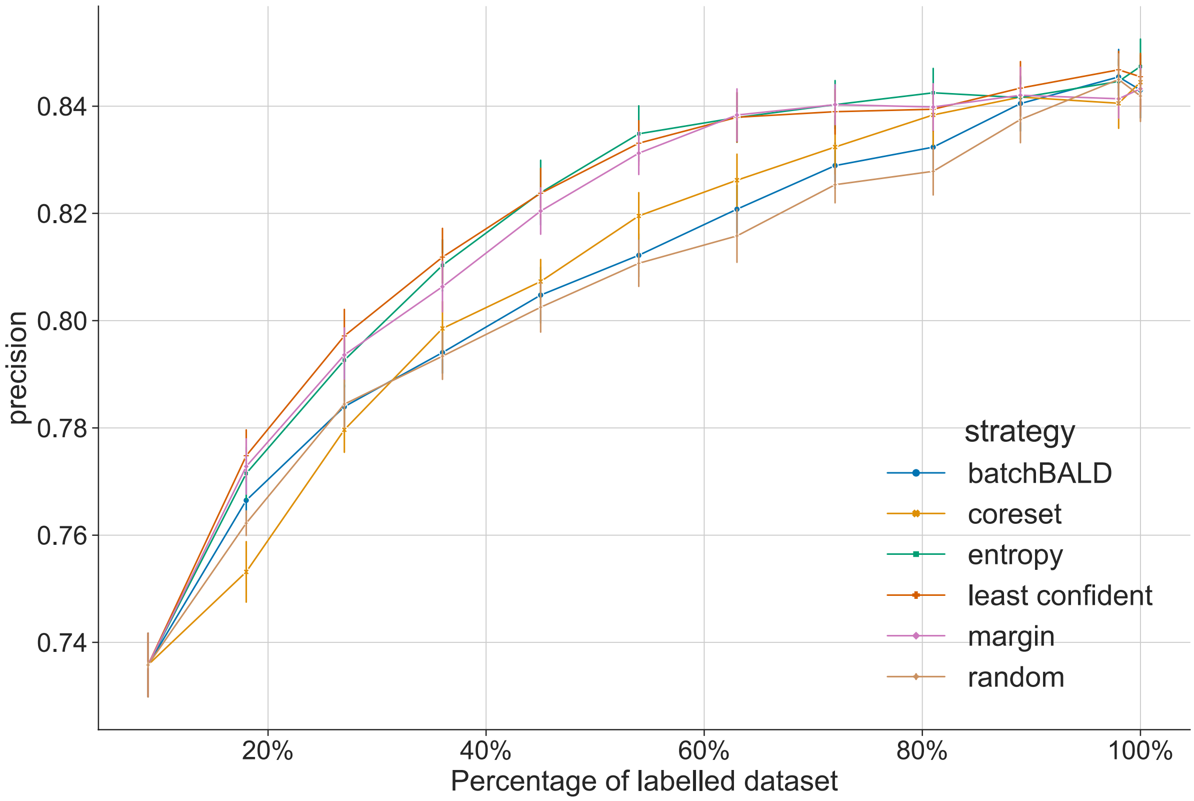

S30 Fig. Precision over the active learning iterations for the Nary-DGV data set. Error bars correspond to the standard error.

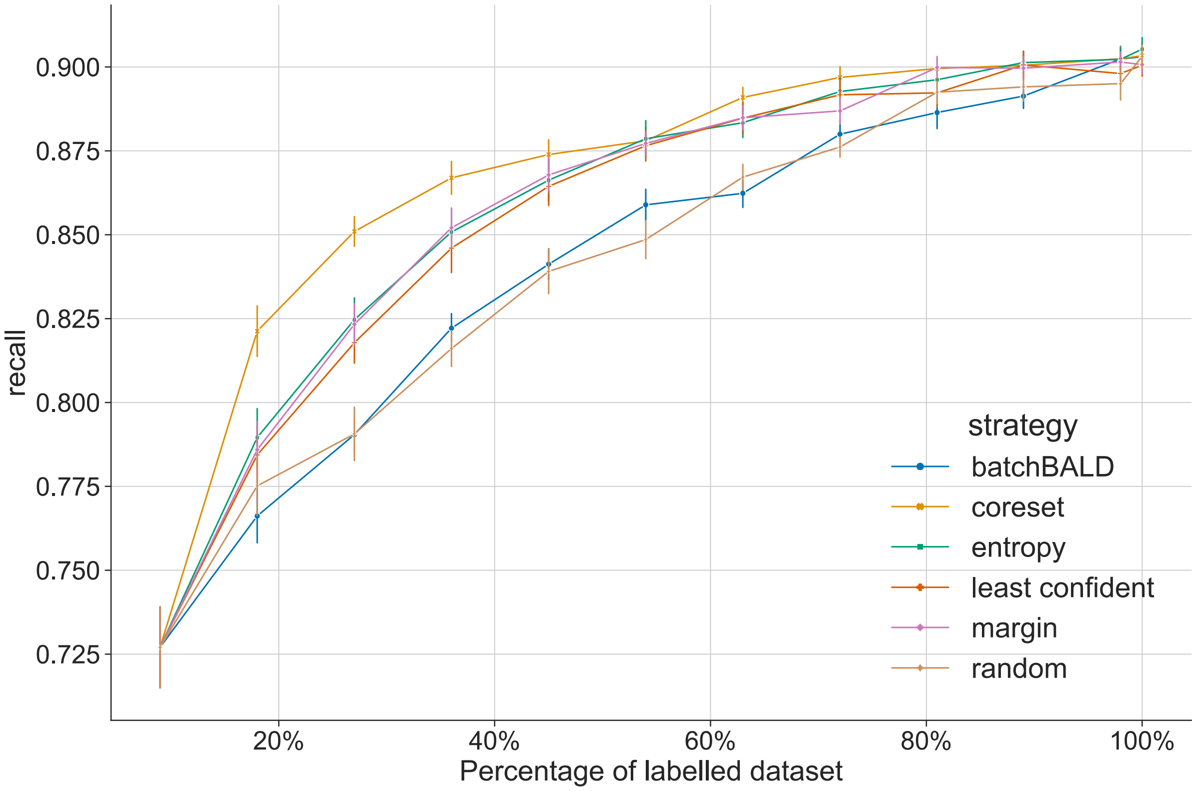

S31 Fig. Recall over the active learning iterations for the Nary-DGV data set. Error bars correspond to the standard error.

Nary-DV

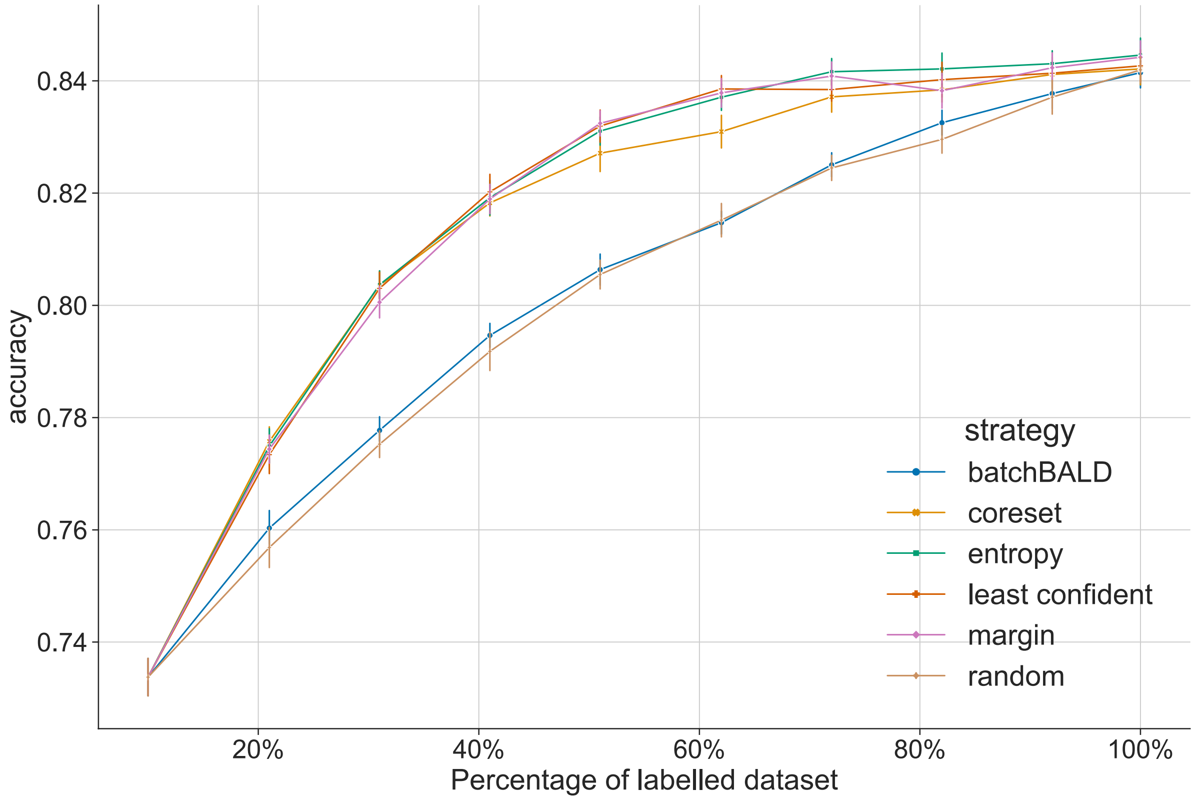

S32 Fig. Accuracy over the active learning iterations for the Nary-DV data set. Error bars correspond to the standard error.

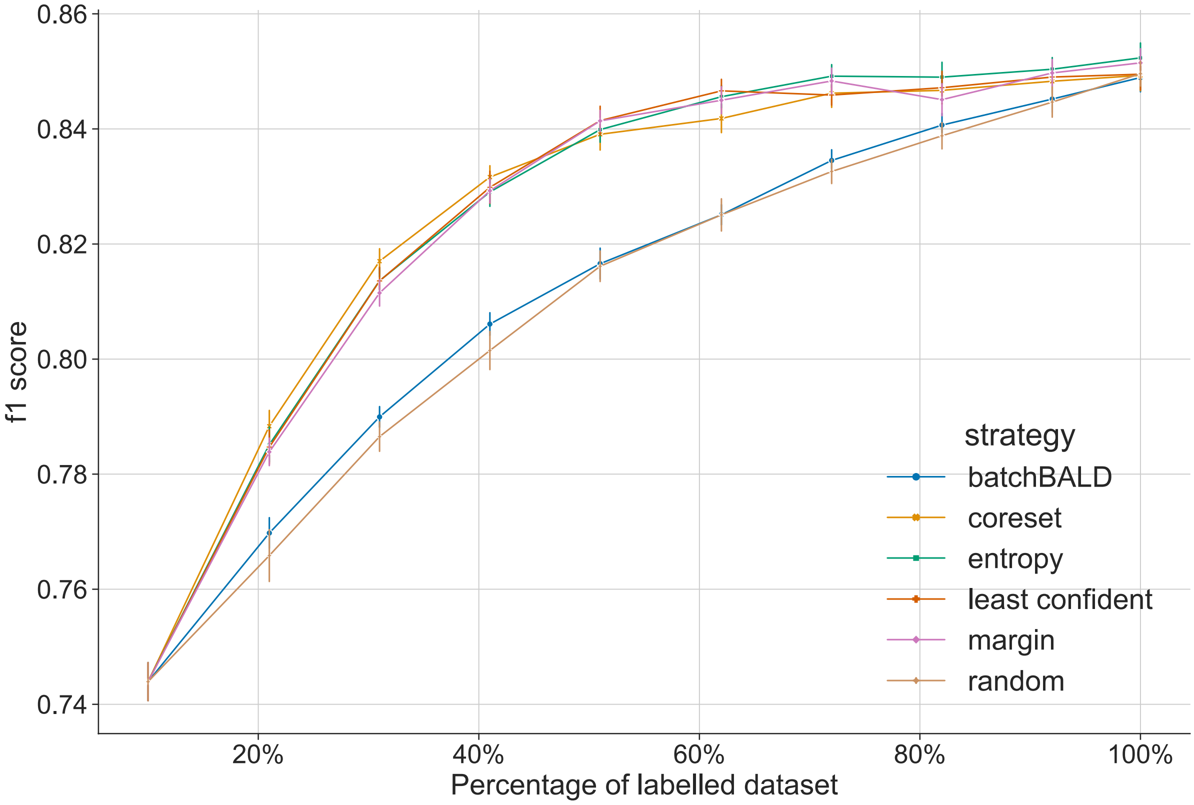

S33 Fig. F1-score over the active learning iterations for the Nary-DV data set. Error bars correspond to the standard error.

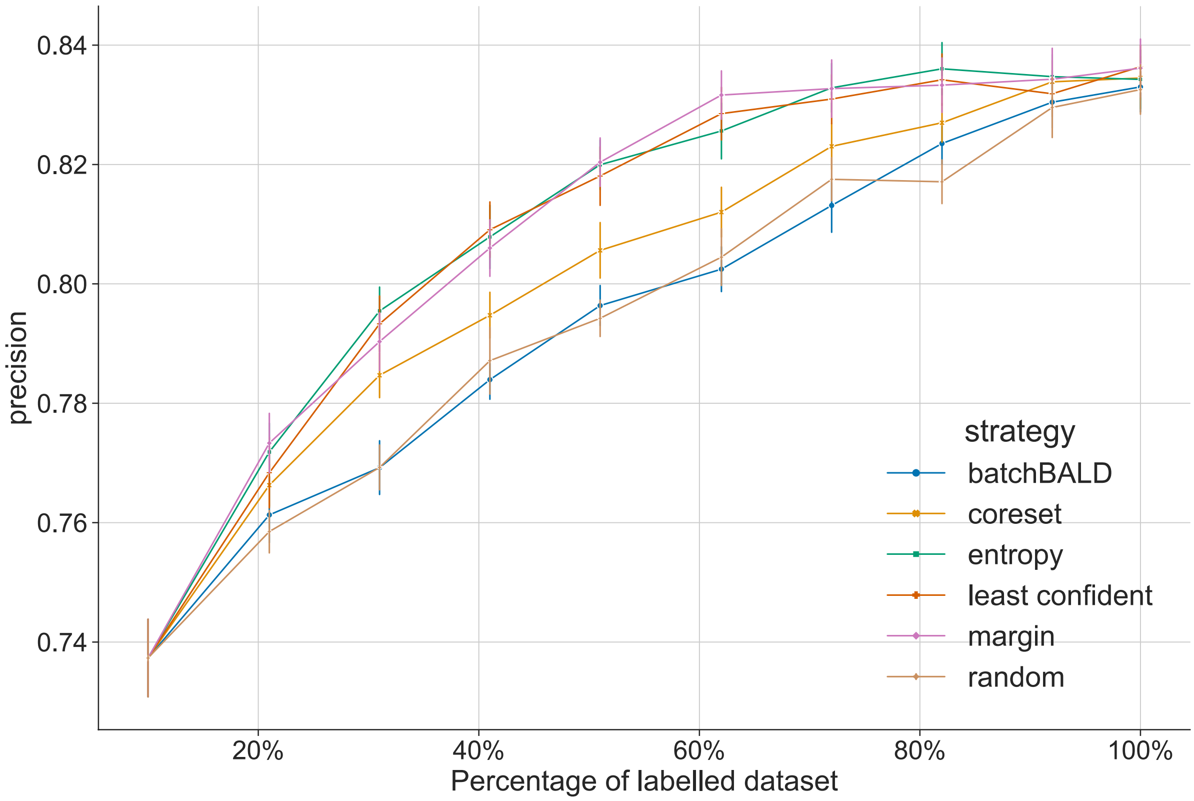

S34 Fig. Precision over the active learning iterations for the Nary-DV data set. Error bars correspond to the standard error.

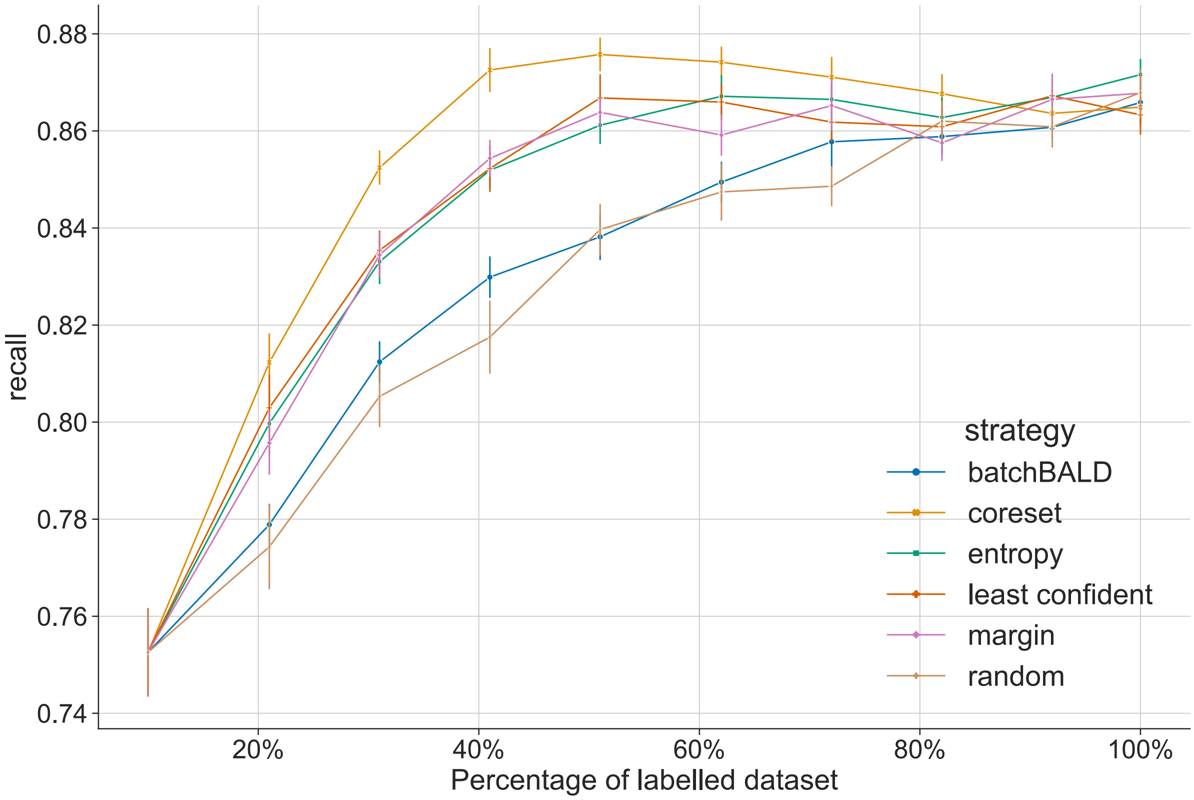

S35 Fig. Recall over the active learning iterations for the Nary-DV data set. Error bars correspond to the standard error.
