# Supplementary material for: A study of deep active learning methods to reduce labelling efforts in biomedical relation extraction
Source: PLoS One. 2023 Dec 15;18(12):e0292356. doi: 10.1371/journal.pone.0292356 (PMC10723703; doi:10.1371/journal.pone.0292356)
Supplement: S3 File — (PDF) [file pone.0292356.s003.pdf]

Supplementary File 3 for

A Study of Deep Active Learning Methods to Reduce Labelling Efforts in Biomedical Relation Extraction

Charlotte Nachtegael, Jacopo De Stefani, Tom Lenaerts

Corresponding author:
Charlotte Nachtegael: Charlotte.Nachtegael@ulb.be

This PDF file includes:
Supplementary Figures 37 to 41

Supplementary Figures

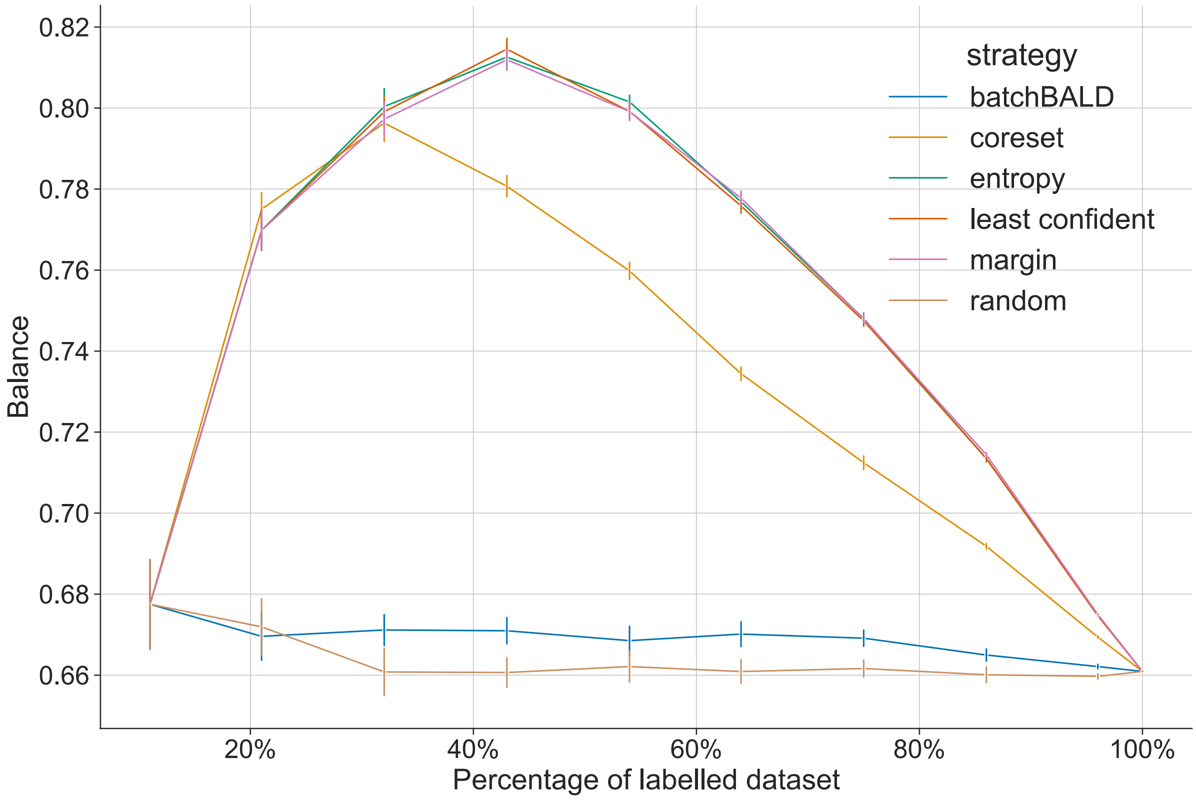

S37 Fig. Balance measures based on Shannon entropy across active learning iterations for the AIMED data set. Error bars correspond to the standard error.

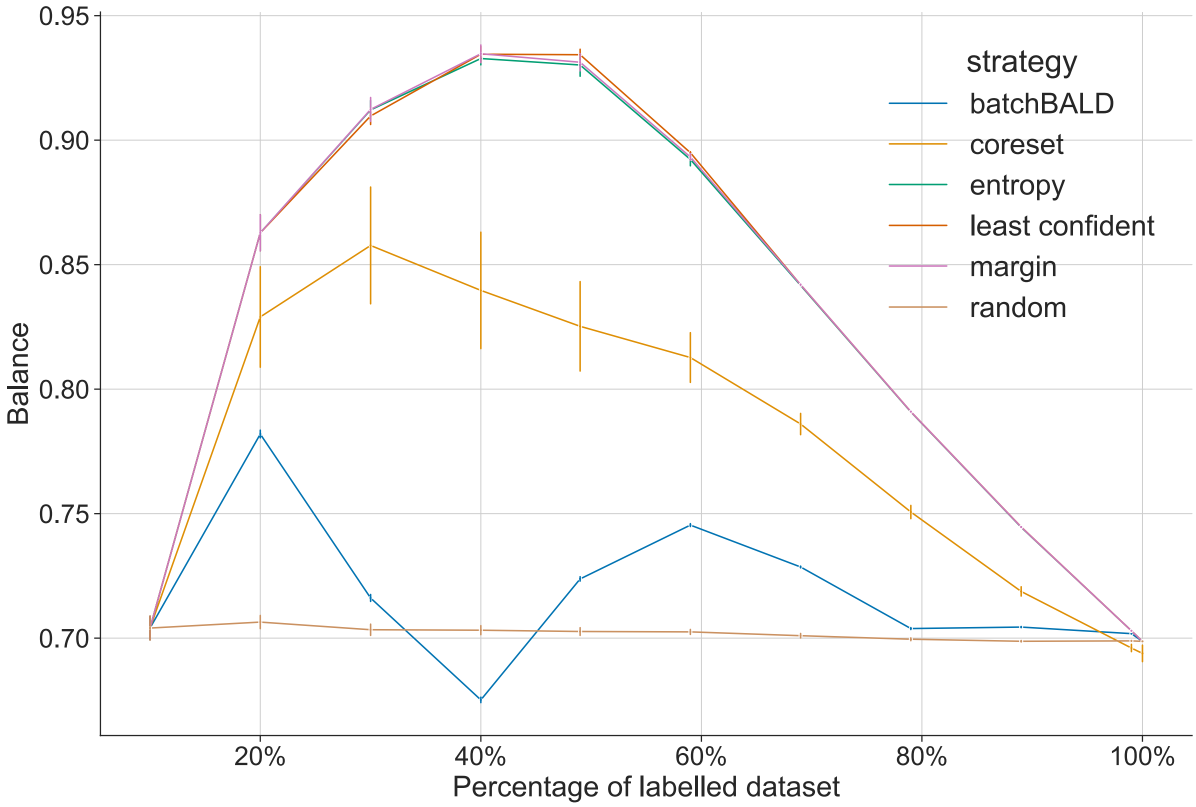

S38 Fig. Balance measures based on Shannon entropy across active learning iterations for the BioREDdata set. Error bars correspond to the standard error.

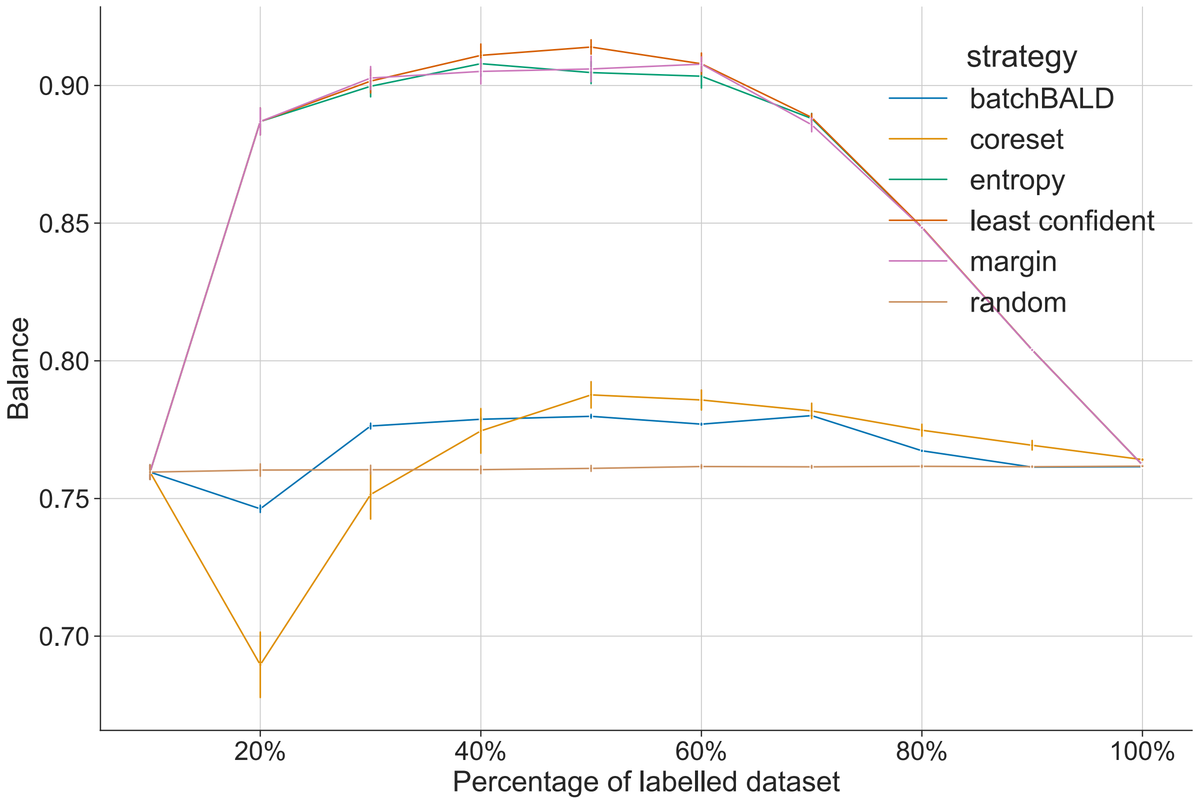

S39 Fig. Balance measures based on Shannon entropy across active learning iterations for the ChemProt data set. Error bars correspond to the standard error.

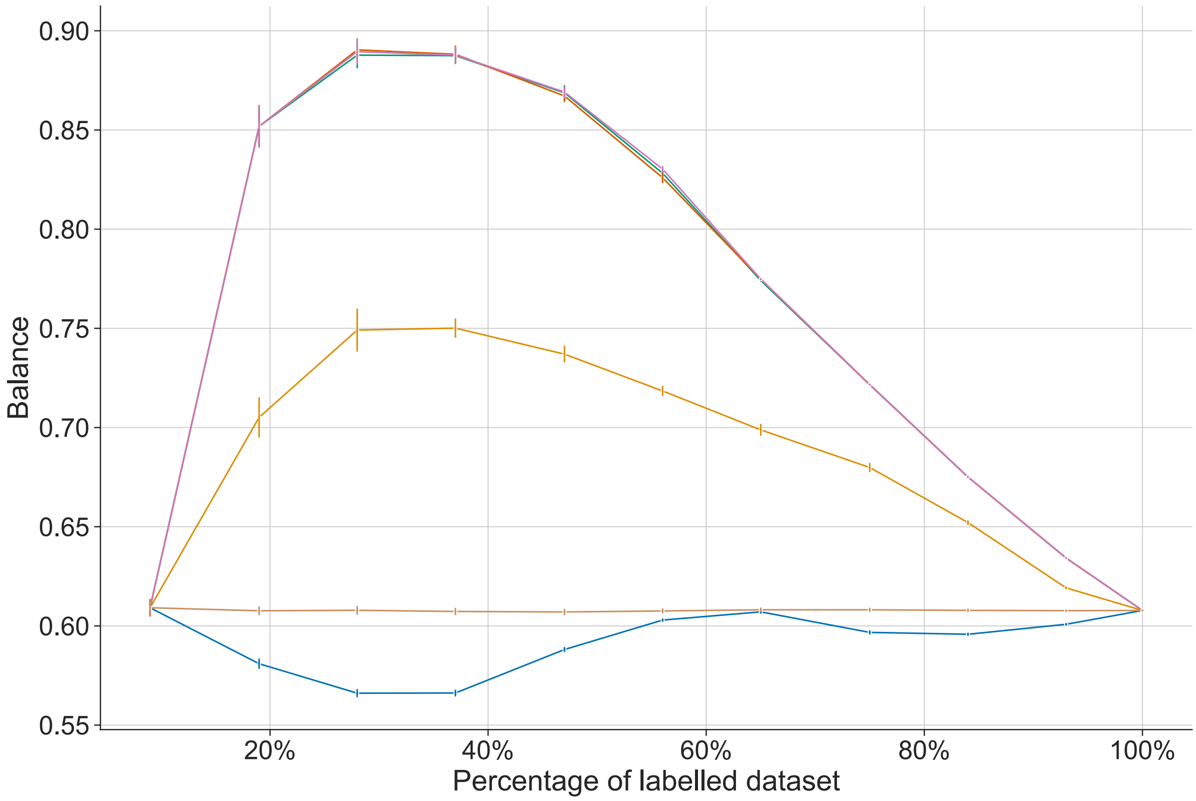

S40 Fig. Balance measures based on Shannon entropy across active learning iterations for the DDI data set. Error bars correspond to the standard error.

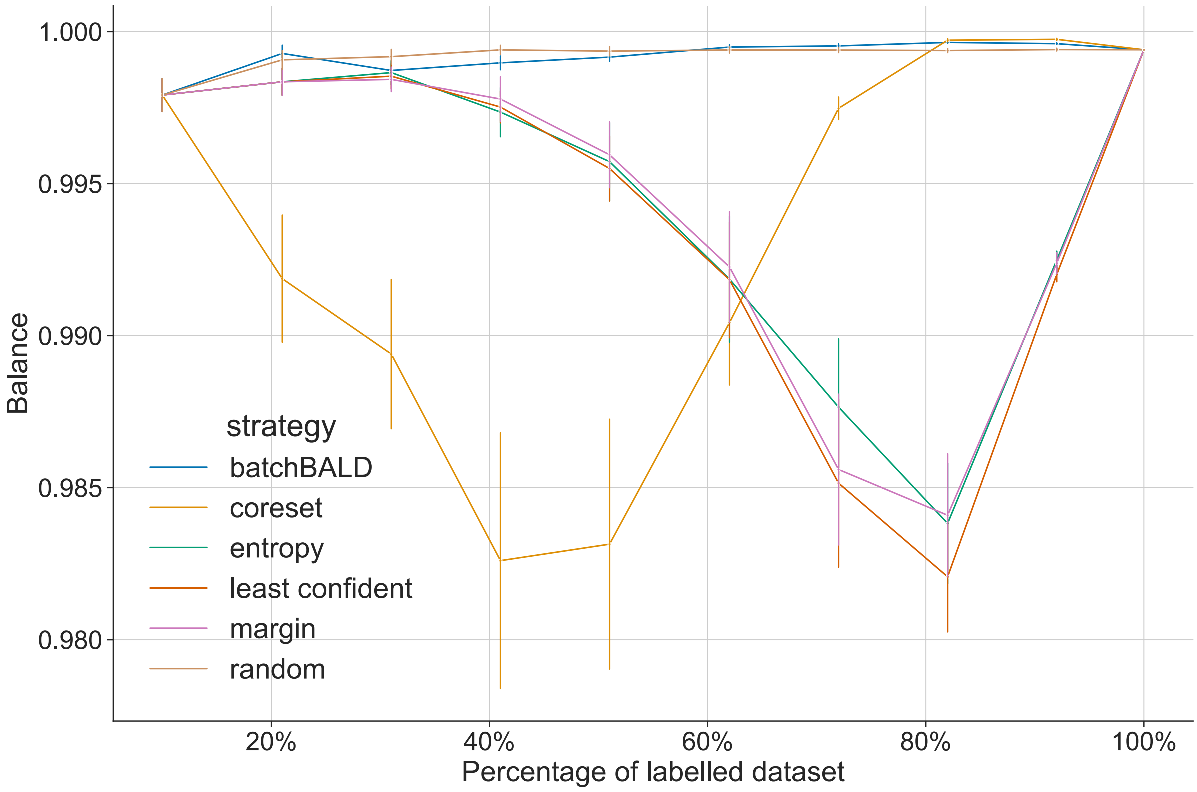

S41 Fig. Balance measures based on Shannon entropy across active learning iterations for the Nary-DV data set. Error bars correspond to the standard error.
