# Supplementary material for: A study of deep active learning methods to reduce labelling efforts in biomedical relation extraction
Source: PLoS One. 2023 Dec 15;18(12):e0292356. doi: 10.1371/journal.pone.0292356 (PMC10723703; doi:10.1371/journal.pone.0292356)
Supplement: S1 Table — Dominant strategies are in the row labels, i.e. the hypothesis tested is the strategy in the row performs better than the strategy in the column. P-values below 0.05 are in bold. (DOCX) [file pone.0292356.s004.docx]

|  | batchBALD | Core-set | Entropy | Least confident | Margin | Random |
| --- | --- | --- | --- | --- | --- | --- |
| batchBALD |  | 1.0 | 1.0 | 1.0 | 1.0 | 0.996 |
| Core-set | **< 0.001** |  | 1.0 | 1.0 | 0.951 | **0.013** |
| Entropy | **< 0.001** | **< 0.001** |  | 0.349 | 0.079 | **< 0.001** |
| Least confident | **< 0.001** | **< 0.001** | 0.660 |  | **0.022** | **< 0.001** |
| Margin | **< 0.001** | 0.052 | 0.924 | 0.980 |  | **< 0.001** |
| Random | **0.005** | 0.988 | 1.0 | 1.0 | 1.0 |  |

**Table S1. Page trend statistical test with intermediate results based on accuracy.** Dominant strategies are in the row labels, i.e the hypothesis tested is the strategy in the row performs better than the strategy in the column. P-values below 0.05 are in bold.
